# An atlas of protein turnover rates in mouse tissues

**DOI:** 10.1038/s41467-021-26842-3

**Published:** 2021-11-26

**Authors:** Zach Rolfs, Brian L. Frey, Xudong Shi, Yoshitaka Kawai, Lloyd M. Smith, Nathan V. Welham

**Affiliations:** 1 Department of Chemistry, University of Wisconsin-Madison, Madison, WI 53706 USA; 2 Division of Otolaryngology–Head and Neck Surgery, Department of Surgery, University of Wisconsin School of Medicine and Public Health, Madison, WI 53792 USA; 3 Present Address: Department of Otolaryngology–Head and Neck Surgery, Graduate School of Medicine, Kyoto University, Kyoto, 606-8507 Japan

**Keywords:** Proteomic analysis, Proteomics, Mass spectrometry, Proteome informatics

## Abstract

Protein turnover is critical to cellular physiology as well as to the growth and maintenance of tissues. The unique synthesis and degradation rates of each protein help to define tissue phenotype, and knowledge of tissue- and protein-specific half-lives is directly relevant to protein-related drug development as well as the administration of medical therapies. Using stable isotope labeling and mass spectrometry, we determine the in vivo turnover rates of thousands of proteins—including those of the extracellular matrix—in a set of biologically important mouse tissues. We additionally develop a data visualization platform, named ApplE Turnover, that enables facile searching for any protein of interest in a tissue of interest and then displays its half-life, confidence interval, and supporting measurements. This extensive dataset and the corresponding visualization software provide a reference to guide future studies of mammalian protein turnover in response to physiologic perturbation, disease, or therapeutic intervention.

## Abstract (web summary)

Protein turnover underpins biology but is challenging to measure in vivo across the entire proteome. Here, the authors provide a comprehensive resource of protein turnover in mouse tissues and develop a visualization platform to analyze these data.

## Introduction

Protein turnover is a fundamental process in all living organisms. Cells are continually creating and destroying proteins to maintain proteostasis. The rate of protein turnover can change in response to physiologic stimuli^1^, development and aging^2^, and disease^3^. Information about each protein’s turnover rate is also relevant to medical and surgical therapies because protein turnover impacts pharmacokinetics and pharmacodynamics^4^, as well as tissue remodeling during wound healing^5^ and following graft transplantation^6^.

Most techniques for measuring protein turnover are limited to bulk determination of average turnover rate in a cell population or tissue, or individual analysis of a small number of isolated proteins^7–10^. Simultaneously measuring individual protein turnover rates for thousands of proteins requires the broad identification and quantification capabilities of state-of-the-art proteomics. Mass spectrometry-based proteomics typically and capably performs proteome-wide identification and quantification at a snapshot in time^11^, whereas measuring proteome synthesis rates, degradation rates, or combined turnover rates comprise a challenging subfield^12,13^. For steady-state protein turnover studies, stable isotope labels are introduced to the organism of interest and proteins sampled over time to monitor the rate of stable isotope incorporation. This method is most feasible for cultured cells^14–19^ and tissues in vitro^6^, where the cost of isotopically-labeled media is modest and serial sampling can occur without necessarily sacrificing the entire culture. In contrast, stable isotopic labeling of mammalian tissues in vivo requires expensive isotopically-labeled food or water, and the collection of many tissues requires sacrificing an organism for each measurement.

In vivo, isotopic labeling can be achieved through different strategies. Some studies have utilized heavy water (^2^H_2_O) in mice and humans (with sampling of blood plasma)^20,21^, while others have administered ^15^N-enriched food to mice and rats^22–26^. Although these methods can yield turnover rates across the proteome, their resulting data quality and analysis are hindered by inherently broad isotope distributions due to variable numbers of heavy atoms present in the proteins. Analytically, the most robust labeling strategy employs a diet containing a labeled amino acid, such as the ^13^C_6_, ^15^N_2_-lysine used in the present work. This auxotrophic, abundant, metabolically-isolated amino acid (lysine) with a large, unique mass shift (8 Da) and a corresponding protease (Lys-C) greatly minimizes the increase in spectral complexity associated with isotopic labeling^7^. Only a few studies have measured protein turnover in mammals using isotopic labeling with a heavy amino acid^27–32^ and, to our knowledge, there is no publicly available dataset containing proteome-wide protein turnover measurements across multiple mammalian tissues.

We have prepared a resource of in vivo protein turnover rates across the proteome for representative mammalian tissues from young adult mice. Half-lives for over 3000 unique proteins were determined, providing an important reference for understanding mammalian biology and an aid for the development of therapeutic proteins or therapies directed against specific protein targets^33^. We developed a software program, named ApplE Turnover (**Application for Elucidating Protein Turnover**)^34^, to calculate half-lives from the experimental data and greatly enhance the accessibility of this resource. This program allows global visualization of half-lives for each tissue, as well as a search function for individual peptides and proteins. The user is thus able to search for a given protein of interest and immediately visualize the half-life along with the extent and confidence of protein turnover evidence. The ApplE Turnover software program and the data results files for each tissue have been made freely and publicly available through the MassIVE repository (http://massive.ucsd.edu) using the dataset identifier MSV000086426, and a tutorial for users has been provided (Supplementary Note 1).

## Results

### Overview

**Fig. 1:**
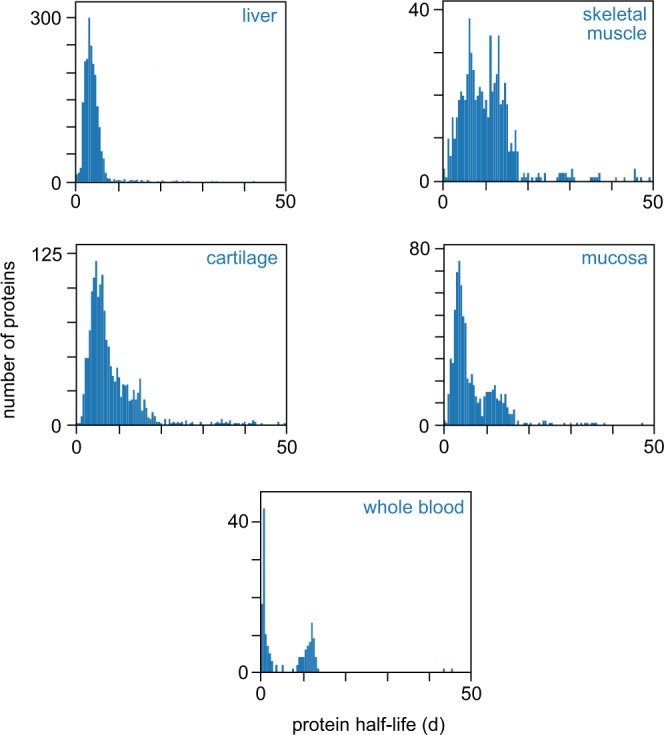
Protein half-life distributions for each tissue class in this resource. Histograms are output by ApplE Turnover. Source data are provided with this paper; raw mass spectrometry data are available as described in the “Data Availability” section.

We analyzed five distinct tissue classes to determine protein turnover rates across the proteome. The five tissue classes (liver, skeletal muscle, cartilage, mucosa, and blood) were chosen because they each contain specialized constituent cells and matrices, have diverse biologic functions, and are morphologically distinct. Liver is an accessory digestive organ with key metabolic functions, skeletal muscle is a contractile tissue, cartilage is a structural component of the skeletal system, mucosa is an immunologically active barrier tissue that interfaces with the external environment, and blood is a circulating biofluid that perfuses other tissues. These diverse tissue types encompass a wide range of protein turnover rates and enable comparisons across classes (Fig. 1). Comparison of different tissues within classes is also possible, as two muscle and three cartilage sites were analyzed, for a total of eight tissues.

The data used to calculate protein turnover rates were generated from mice that contained ~99% isotopically-labeled lysine (Lys8) and were switched to a diet containing unlabeled lysine (Lys0) on day zero; tissues were harvested 3, 7, 14, 30, and 60 d following removal of the isotopically labeled food (Online Methods–Mice). A control experiment showed that the direction of isotopic change (i.e., Lys0-to-Lys8 versus Lys8-to-Lys0) does not impact the half-life measurements (Supplementary Fig. 1), as reported by others^7,25^. Proteins were extracted from the mouse tissues, digested into peptides with endoproteinase Lys-C, and analyzed by mass spectrometry-based proteomics. The data were processed to identify and quantify both the labeled (Lys8-containing) and unlabeled (Lys0-containing) versions of each peptide. These data served as input for the ApplE Turnover software to calculate peptide half-lives and the associated protein half-lives.

**Fig. 2:**
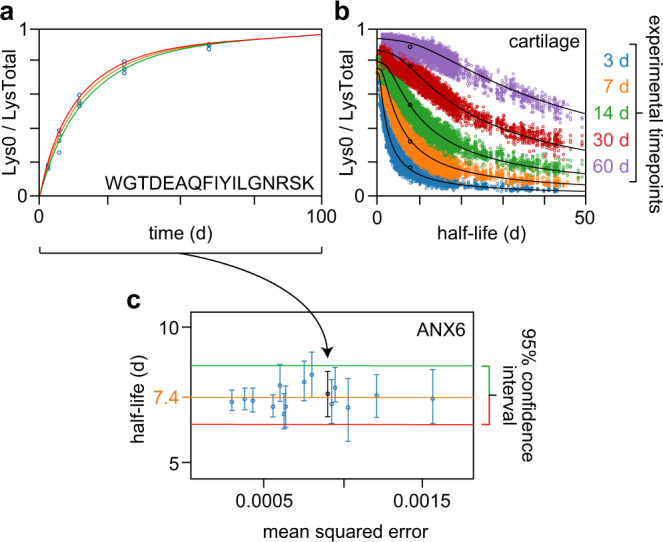
Example output plots from ApplE Turnover. **a** Experimental data (open blue circles) collected at the 3, 7, 14, 30, and 60 d timepoints for the peptide WGTDEAQFIYILGNRSK from the protein ANX6 in cartilage. The *y* axis indicates the relative fraction of this peptide that contains unlabeled (i.e., new) lysine (Lys0/LysTotal, where LysTotal is the sum of Lys0 and Lys8 quantities). ApplE Turnover uses these data to generate a model fit (orange curve) and 95% confidence interval (bound by the red and green curves). **b** Data for all quantified peptides in cartilage. Black curves indicate the model fits for this tissue; black open circles indicate the mean relative lysine fractions for the WGTDEAQFIYILGNRSK peptide at its calculated half-life of 7.5 d. **c** Half-lives for ANX6-derived peptides in cartilage plotted as a function of mean squared error (of the model fit); the WGTDEAQFIYILGNRSK data are black and indicated by an arrow. The calculated half-life for ANX6 is 7.4 d (orange line); the 95% confidence interval is bound by the red and green lines. The peptide half-life data are plotted as the best model fit to all observations of a given peptide ± the 2.5th and 97.5th percentile values from 200 simulations per peptide, based on a minimum of six peptide observations (of which at least three occurred at a single experimental timepoint), from a pool of *n* = 22 biologically independent samples. Source data are provided with this paper; raw mass spectrometry data are available as described in the “Data Availability” section.

ApplE Turnover uses the data from thousands of peptides in a tissue to calculate tissue-specific protein turnover rates. Data for an example peptide are shown in Fig. 2a: its relative fraction of unlabeled (i.e., new) lysine (Lys0/LysTotal) increases over time, as expected. The software employs a three-compartment model^23^ (Methods–ApplE Turnover) that considers both the delay in availability of newly-introduced unlabeled lysine amino acids after switching the mice to unlabeled food, as well as the recycling of labeled lysine amino acids after catabolism of old label-containing proteins. The model assumes that unlabeled proteins must have been synthesized after the switch to the unlabeled-lysine diet. In contrast, it does not assume that all labeled proteins were synthesized before the diet switch, because previously-existing labeled proteins are continually undergoing degradation and thereby provide a recycled source of labeled lysine to ribosomes for new protein synthesis.

The three compartments in this model—where unlabeled and labeled lysines can reside—are the pool of free amino acids, the pool of all proteins, and the pool of the protein of interest. The kinetic coefficients for transfer between compartments are determined using nonlinear regression of the proteome-wide Lys0 and Lys8 peptide abundance data (Online Methods–ApplE Turnover). These coefficients are unique to each tissue, thereby providing insight into how quickly a given tissue receives access to new free lysine, as well as the extent of lysine recycling from degraded proteins. From these coefficients, we can predict the relative fraction of available unlabeled lysine at any point in time. This information, along with the observed peptide-specific values for the relative fraction of unlabeled-lysine-containing peptide at each timepoint (i.e., Lys0/LysTotal, as shown in Fig. 2a), allows for the calculation of that peptide’s half-life in that tissue. All peptide half-lives and the timepoint data from which they were calculated are displayed for cartilage in Fig. 2b. The black curves show a satisfactory overall fit of the model to the proteomic data in this tissue. The peptide half-lives corresponding to a given protein are then used to determine that protein’s half-life, as shown in Fig. 2c for the example protein Annexin A6 (ANX6) in cartilage.

Protein half-lives were determined for 3,106 unique proteins. Many of these proteins were identified and quantified across multiple tissues to yield a total of 8,319 protein half-lives. Substantially more unique proteins (5,413) and peptides (76,687) were identified in this reference set than could be quantified precisely enough to report half-life values (Supplementary Table 1). Quantification generally requires higher data quality than does identification, including for mass spectrometry-based proteomics of very complex samples. The likelihood of determining a peptide half-life strongly correlates with the observed intensity of that peptide (Supplementary Fig. 2). The half-life distributions for the proteins that were quantified in each tissue class are shown in Fig. 1. These half-lives spanned a wide range, from less than one day to hundreds of days. The median half-life for each tissue ranged from 3.6 d for liver to 10 d for skeletal muscle. The median uncertainty in each half-life measurement across all tissues, estimated using 95% confidence intervals, was ±0.9 d.

### Tissue-specific differences

**Fig. 3:**
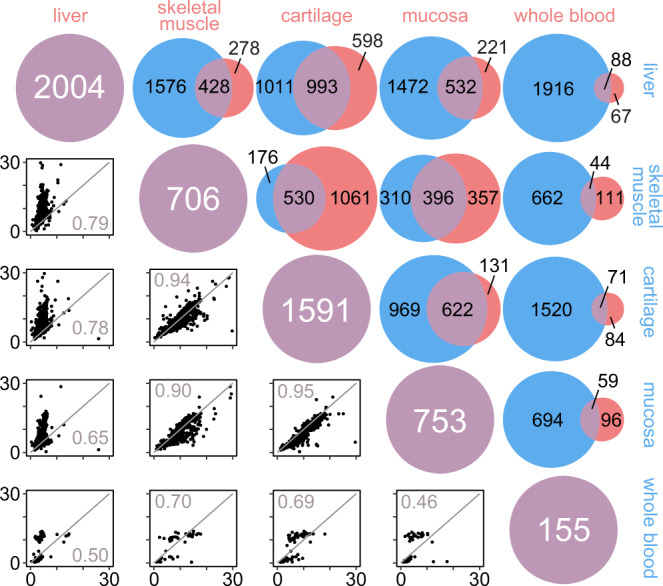
Comparison of protein half-life measurements among all tissue classes in this resource. Venn diagrams show the number of protein half-lives determined for each tissue (blue circles depict tissues listed at the right; red circles depict tissues listed above; purple circles indicate the total number of half-lives for each tissue) and the number of proteins with half-lives that are shared between tissues (purple intersections). Scatterplots show the correlation of half-lives (d) for shared proteins in each paired comparison; an identity line (*y* = *x*) and correlation coefficient (Pearson’s *r*) are superimposed on each plot. Source data are provided with this paper; raw mass spectrometry data are available as described in the “Data Availability” section.

Protein half-lives appear to be largely conserved across tissues, but there are notable differences. The correlation plots in Fig. 3 show that liver proteins turn over much faster than identical proteins in other tissues, in agreement with prior work characterizing average bulk protein turnover in liver^35^. In contrast, skeletal muscle proteins turn over somewhat more slowly. For example, 393 (92%) of the 428 protein half-lives found in both liver and muscle had a longer half-life in muscle, and 229 (54%) of them had a half-life more than twice as long. All three cartilage tissues had similar protein half-lives and substantial overlap of the proteins whose half-lives could be determined, suggesting that protein turnover rates are conserved among similar tissues (Supplementary Fig. 3). This also appears to be largely true for the two muscle tissues; however, we observed that some proteins (notably those associated with contractile, metabolic, and regenerative functions) turned over faster in intrinsic laryngeal muscle than sternocleidomastoid (Supplementary Fig. 4), consistent with the energy-demanding superfast phenotype of these laryngeal muscles for airway protection and vocalization^36^.

**Fig. 4:**
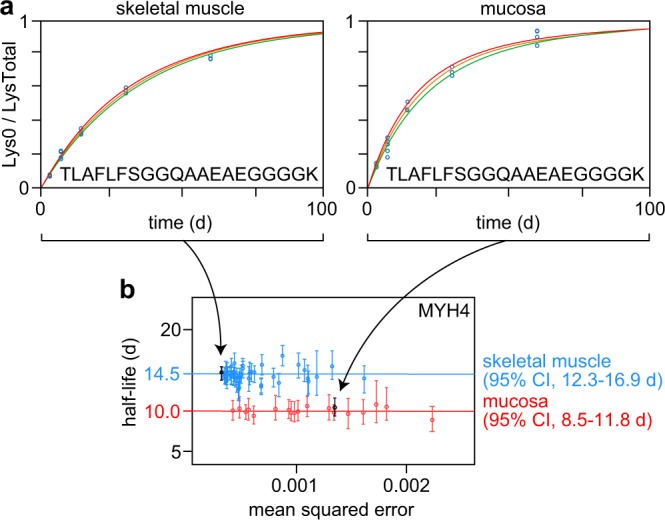
Comparison of differential half-lives for an example protein in two tissues using plots output by ApplE Turnover. **a** Relative lysine fractions (open blue circles) for the peptide TLAFLFSGGQAAEAEGGGGK from the protein MYH4 in skeletal muscle and mucosa. The respective model fits (orange curves) and 95% confidence intervals (bound by the red and green curves) are shown. **b** Half-lives for MYH4-derived peptides in skeletal muscle (blue) and mucosa (red) plotted as a function of mean squared error (of the model fit); the TLAFLFSGGQAAEAEGGGGK data are black and indicated by an arrow. The calculated half-lives for MYH4 in skeletal muscle and mucosa are indicated by blue and red horizontal lines, respectively. The peptide half-life data are plotted as the best model fit to all observations of a given peptide ± the 2.5th and 97.5th percentile values from 200 simulations per peptide, based on a minimum of six peptide observations (of which at least 3 occurred at a single experimental timepoint), from a pool of *n* = 22 biologically independent samples. Source data are provided with this paper; raw mass spectrometry data are available as described in the “Data Availability” section.

Most proteins have similar half-lives among tissues, but researchers may be interested in those with statistically significant differences between tissues. ApplE Turnover calculates *P*-values and fold changes for the half-lives of each protein observed in multiple tissues and allows for easy visualization of the data supporting each difference (Fig. 4). Researchers can use the built-in search box to easily find their protein of interest, and then display all peptide half-lives for that protein to show the distribution and confidence of turnover rates for separate tissues. Figure 4 shows the supporting information for the half-lives reported for Myosin-4 (MYH4), a motor protein and component of the skeletal muscle contractile apparatus. These data confidently show that MYH4 turns over more slowly in muscle than in mucosa.

### Blood proteins

The resource uncovered protein turnover characteristics that appear unique to blood and its function within the circulatory system and in perfused tissues. First, the protein half-lives for blood exhibit a clear bimodal distribution (Fig. 1). The most abundant short (<3.5 d) and long (>3.5 d) half-life proteins are albumin and hemoglobin, respectively, indicating that the bifurcation in half-lives might reflect protein sets attributable to the primary components of whole blood: plasma and cells (most of which are hemoglobin-carrying erythrocytes). We assessed this possibility further by reviewing the two protein sets and found that 66 of 85 (78%) of the shorter half-life proteins have a secreted designation in UniProt, compared to 8 of 70 (11%) of the longer half-life proteins. A gene ontology (GO) analysis—performed on each protein set using the other set as background (Supplementary Data 1)—corroborated the plasma designation for the shorter half-life set by identifying extracellular proteins associated with regulation of various catalytic activities, defense response, and humoral immunity; in contrast, the longer half-life set was comprised of intracellular proteins involved in metabolic and biosynthetic processes, along with oxidoreduction and other catalytic functions.

Although the bimodal distribution of protein half-lives in whole blood is satisfactorily explained by distinct turnover rates for circulating plasma and cells, we caution that these half-life estimates may be less precise than those reported for solid tissues. This is because the model assumes that proteins undergo synthesis, lifetime residence, and degradation solely within the analyzed tissue. This assumption does not hold for blood, however, as plasma proteins are predominantly synthesized in the liver and secreted into circulation; and blood cells arise in bone marrow, enter circulation, and are eventually degraded in the liver and spleen. Future studies might improve measurement precision and half-life estimates somewhat by fractionating blood prior to proteomic analysis. Even then, additional model development is required to accurately reflect amino acid recycling and the relative amounts of heavy and light isotopic amino acids available in the multiple biologic compartments in which blood proteins are synthesized.

The issue of blood protein synthesis occurring outside of the analyzed tissue also has a small impact upon turnover estimates in the solid tissues. We detected little hemoglobin in these tissues, consistent with effective transcardial perfusion of the vasculature with buffer solution at the time of harvest, but we did identify plasma proteins that presumably entered the tissues by diffusion. Inspection of the model curve fits for abundant plasma proteins within the solid tissues reveals that some data points lie in an impossible region above the curve representing free lysine turnover. Supplementary Fig. 5 illustrates this phenomenon for skeletal muscle albumin. Consider a hypothetical protein with an instantaneous half-life (*t*_1/2_ = 0): the relative fraction of Lys0 in this protein would match that of free amino acid Lys0 over time due to immediate and repeated degradation and resynthesis, with each synthesis event drawing from the available free lysine pool. However, the example in Supplementary Fig. 5 shows an albumin turnover rate that exceeds that of free lysine in skeletal muscle, in violation of the model assumptions. The explanation for this discrepancy between the data and model-predicted maxima is that albumin is not synthesized in skeletal muscle, but exogenously with access to a separate amino acid pool. Therefore, caution is required when interpreting half-life values for exogenous proteins. Such exogenous blood proteins represent only 2–4% of all half-life estimates across the solid tissues in the dataset, and therefore have minimal impact on the determinations of individual tissue uptake/recycling coefficients and the endogenous protein turnover measurements.

### Long-lived proteins

**Fig. 5:**
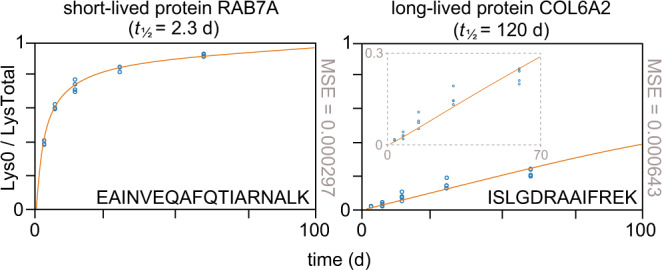
Differences in model fit for short- and long-lived proteins. Relative lysine fractions (open blue circles) and model fits (orange curves) for peptides from representative short- (RAB7A) and long-lived (COL6A2) proteins. The mean squared error (MSE) for each model fit is shown to the right of each plot. The model fits well to most peptide data, as illustrated for RAB7A, but fits poorly to long-lived proteins. In such cases, as shown for COL6A2, the model tends to underestimate Lys0 incorporation at 14 and 30 d, while overestimating it at 60 d (see inset). One explanation for this observation is that only a fraction of the long-lived protein is turning over while the remainder is static. Source data are provided with this paper; raw mass spectrometry data are available as described in the “Data Availability” section.

Most proteins were found to turn over in a matter of days, but some long-lived proteins have half-lives on the scale of months or years. These long-lived proteins are of particular relevance to aging and degenerative diseases^25,37–40^. Such proteins were identified in all solid tissues but were most prevalent in cartilage. GO analysis of cartilage proteins with half-lives greater than 21 d implicated these proteins in cell adhesion, morphogenesis, and the extracellular matrix (Supplementary Data 2). These GO terms are representative of structural proteins, such as collagens, which are expected to have long half-lives^25,41^. A notable feature of these long-lived proteins is their relatively poor fit to the three-compartment model, compared to the short-lived proteins (Fig. 5). Our model assumes that all proteins are available for degradation and that degraded proteins are continually being replaced by newly synthesized proteins. However, we observed that a fraction of each long-lived protein was not degraded and remained labeled with Lys8, at least for the 60-d time course of the experiment. This observation directly impacts the model fit: whereas the time series data appear to form an asymptote in Lys0 incorporation for many long-lived proteins, the model fails to fit this asymptote and continues to rise.

Special consideration should be taken when using the reported half-lives for these long-lived proteins. Such cases are readily identifiable by their long half-lives, poor fits to the model, and consequent wide confidence intervals. A possible biologic contributor to inaccurate half-life estimates for the long-lived proteins in our dataset is late postnatal tissue development. The mice used in this study were 8–10-week-old young adults, whereas mice reach full adult maturity around 12 weeks^42^. Any tissue growth and maturation during this time would result in the rate of protein synthesis exceeding the rate of decay; however, our model assumes a steady-state in protein abundance. Such growth will have a negligible effect when modeling proteins that turn over quickly, but a substantial impact on proteins with very slow rates of decay. If the long-lived proteins identified in this study exhibited much greater synthesis than degradation rates (or were not being degraded at all) at early timepoints, then their reported half-lives are underestimated and, in reality, much longer than as determined by our model. Another possible explanation is that these proteins undergo a non-exponential degradation, whereby they exhibit faster degradation rates in the first few hours of their molecular lifetimes followed by prolonged periods of relative stability^43^.

### Proteoform-specific differences

A single protein can be decorated with post-translational modifications (PTMs) that dramatically alter the function of the protein. These different forms of a given protein are defined as proteoforms and have substantial biological importance^44^. In this study, we were able to identify thousands of peptides containing PTMs and determine their half-lives. Although the identity of an intact proteoform cannot be definitively determined through the identification of a single peptide, the identification of both a modified and unmodified peptidoform is evidence for two distinct groups of proteoforms: one which contains the PTM and one which does not.

**Fig. 6:**
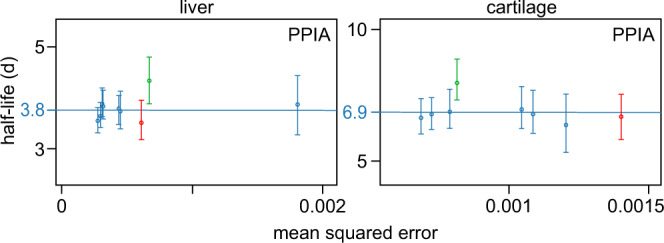
Comparison of proteoform half-lives. Half-lives for PPIA-derived peptides in liver and cartilage, plotted as a function of mean squared error (of the model fit); calculated half-lives for PPIA are indicated by horizontal lines. The N-terminal peptide of PPIA with acetylation (green) exhibits a longer half-life than the non-acetylated form of the same peptide (red), in both liver and cartilage. Other peptides from PPIA (blue) do not contain the N-terminus and so may be from the modified or unmodified proteoform. The peptide half-life data are plotted as the best model fit to all observations of a given peptide ± the 2.5th and 97.5th percentile values from 200 simulations per peptide, based on a minimum of six peptide observations (of which at least three occurred at a single experimental timepoint), from a pool of *n* = 22 biologically independent samples. Source data are provided with this paper; raw mass spectrometry data are available as described in the “Data Availability” section.

We determined half-lives for 3,431 peptides that contained PTMs. Of these 3,431 half-lives, 2,439 had an additional half-life as an unmodified peptidoform. All proteoform group comparisons had their *P*-value and fold change determined by ApplE Turnover. Most PTMs were not found to have a significant influence on protein turnover, but there was a significant difference in 66 cases including acetylation, methylation, phosphorylation, hydroxylation, and carboxylation (Supplementary Data 3). For example, we observed an N-terminal acetylation that appeared to increase the half-life of the protein Peptidylprolyl isomerase A (PPIA) in both liver and cricoid cartilage (Fig. 6). In support of this observation, N-terminal acetylation has previously been shown to influence proteoform half-life^45^. The PTM results reported here highlight the ability of ApplE Turnover to report proteoform-specific differences observed in the half-life data.

## Discussion

This protein half-life dataset and corresponding ApplE Turnover software serve as a powerful resource for studying fundamental protein turnover, interrogating biological questions, and developing therapeutics. We find that the turnover rates for individual proteins can vary significantly across tissue classes, consistent with known differences in tissue composition and physiologic function. We also show that anatomically distinct tissues within a given class have largely similar protein turnover rates. As noted, the three-compartment model does not fully accommodate the physiologic complexity of turnover dynamics for blood and long-lived proteins, warranting caution when interpreting their half-life estimates. However, most protein half-lives (>96%) are robust and useful measures of protein turnover in mammalian tissues with relative confidence intervals below 40% (Supplementary Fig. 6). For example, a protein in this resource with a half-life of 10 d can be reasonably expected to have a 95% confidence interval that spans less than 8–12 d (i.e., 40% of 10 d).

**Fig. 7:**
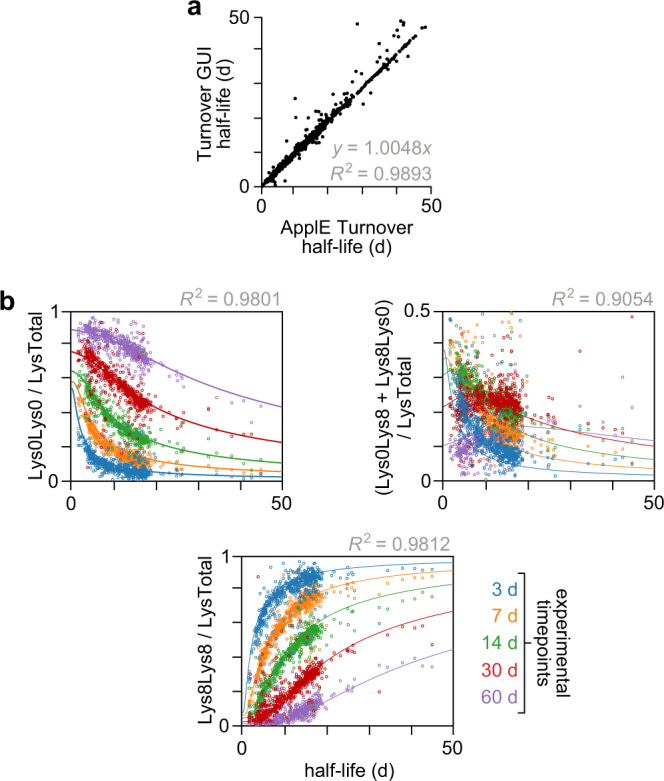
Validation of ApplE Turnover. **a** Comparison of calculated peptide half-lives in cartilage, generated by ApplE Turnover and Turnover GUI. **b** Relative fractions of the three isotopic label combinations (Lys0Lys0, Lys0Lys8, Lys8Lys8) of missed cleavage peptides in cartilage, as a function of peptide half-life. The expected values for each experimental timepoint are plotted as solid curves; the observed data are matched-color open circles. Note that each of these missed cleavage plots is analogous to the single plot in Fig. 2b, which shows the relative fraction of unlabeled lysine (Lys0) for all peptides in cartilage. Results from other tissues are shown in Supplementary Figs. 7 and 8. Source data are provided with this paper; raw mass spectrometry data are available as described in the “Data Availability” section.

Isotopic labeling strategies, such as implemented here, represent a gold standard approach to peptide and protein half-life determination because they allow high-precision measurement of relative abundance with no impact on protein structure. There are, to our knowledge, no methods or datasets for orthogonal validation of proteome-wide half-lives using an independent assay; consequently, this is not a feature of previous labeling studies. Here, to ensure internal and external consistency and precision of the model-based half-life values generated by ApplE Turnover, we pursued a three-pronged validation approach. First, as ApplE Turnover uniquely computes a given protein’s half-life using the half-lives of its constituent peptides (e.g., Fig. 2c), we confirmed consistency across the independently determined peptide half-lives for each protein in the resource (Supplementary Table 2). Second, we validated the peptide and protein half-lives output by ApplE Turnover using a previously published software tool, Turnover GUI^31^. We provided both software programs with identical input in the form of Lys0 and Lys8 peptide quantification data, and the linear regression of their respective half-life values showed robust agreement for all tissue classes (*R*^2^ = 0.9876–0.9973; Fig. 7a and Supplementary Fig. 7).

Third, we conducted further validation of our half-life measurements, as well as assumptions underpinning the three-compartment model, by analyzing peptides containing two lysine residues. The proteomic data in this resource predominantly arise from peptides containing a single lysine generated by LysC digestion; however, peptides with a missed cleavage contain two lysines and, by extension, one of three isotope label combinations: Lys0Lys0, Lys0Lys8, or Lys8Lys8. Given that ApplE Turnover excludes these missed cleavage peptides from its training set, we used the three-compartment model to predict the relative abundances of Lys0Lys0, Lys0Lys8, and Lys8Lys8 peptides as a function of peptide half-life at each labeling timepoint. The model predictions showed strong agreement with the observed fractional abundances within each tissue class (pooled *R*^2^ = 0.8639–0.9724; Fig. 7b and Supplementary Fig. 8), further validating that the model accurately reflects lysine availability and incorporation within mouse tissues.

In summary, the resource of protein half-lives we describe here provides researchers with insight into the dynamics of protein turnover rates for thousands of proteins across several unique tissue types. Methods for determining comprehensive protein turnover rates in mammalian tissue are cost- and time-prohibitive, and the complexity of tissue proteomics coupled with stable isotope labeling creates a challenge for protein half-life determination. Thus, we have made this resource, including all of the raw and processed data, publicly available, and we have provided a tutorial demonstrating how to access, visualize, and analyze these data (Supplementary Note 1). Furthermore, the tools used to process the raw data (MetaMorpheus, the FlashLFQ algorithm within mzLib, and ApplE Turnover) are written with open-source code to provide analysis transparency and facilitate reproducibility^34,46^. The ApplE Turnover software tool greatly enhances the benefit of the data in this resource, including future additions by us or others, because it enables protein- and tissue-specific searches along with facile data visualization.

## Methods

### Mice

Animal experiments were performed with approval of the Animal Care and Use Committee of the University of Wisconsin School of Medicine and Public Health and complied with all relevant ethical regulations. All mice were housed at 22 °C and 50% relative humidity with a 12 h light/dark cycle, switched at 6 am and 6 pm.

NBSGW mice (Jackson Laboratory stock 026622)^47^ were obtained from the Humanized Mouse Core of the University of Wisconsin-Madison. Heavy isotopically-labeled Lys8–SILAC mouse diet (^13^C_6_, ^15^N_2_-lysine > 98%) was purchased from Silantes (product #230984640; Munich, Germany). Lys8 food was chosen over the Lys6 option to provide additional separation between the peptide isotopic envelopes, which can improve SILAC quantification. The mice were maintained on the Lys8 diet for three generations^48^; Lys8 incorporation was monitored using serial blood draws and mass spectrometry and confirmed to be ~99% at the time of experimentation. The mice had previously undergone subrenal graft implantation, followed by explantation, in an unrelated study that involved no systemic manipulations and had no bearing on the current work. At time zero, *n* = 22 mice (8 male, 14 female) were switched from the labeled Lys8 food to Silantes unlabeled Lys0 food (product #230004600). The mice were 8–10 weeks old at time zero.

Five age-matched and unlabeled C57/BL6J mice (2 male, 3 female; Jackson Laboratory stock 000664) were used as controls to assess: (i) the generalizability of turnover data from the NBSGW strain, and (ii) the impact of the direction of isotopic change. These control mice were raised on a standard unlabeled diet (2018; Teklad), switched to Silantes unlabeled Lys0 food for two weeks, then switched to the Lys8–SILAC diet at time zero. The mice were sacrificed 14 d after the introduction of the heavy isotopically-labeled food and tissues were compared with the 14 d samples from the primary experiment (Supplementary Fig. 1).

### Tissue harvest

NSBGW mice were euthanized by isoflurane overdose 3, 7, 14, 30, and 60 d after the introduction of Lys0 food (*n* = 4–5 per timepoint); as noted above, C57/BL6J mice were euthanized 14 d after the introduction of Lys8 food (*n* = 5). Thirty microliters of whole blood was collected from the retro-orbital plexus, snap-frozen on dry ice, and stored at −80 °C until use. After euthanizing animals and draining blood, mice were additionally perfused with 20 mL of cold PBS to remove residual blood from the circulatory system. Next, liver, skeletal muscle [sternocleidomastoid, intrinsic laryngeal (thyroarytenoid-lateral cricoarytenoid complex)], cartilage (arytenoid, cricoid, thyroid), and mucosa (vocal fold) samples were harvested. Sternocleidomastoid and cricoid are reported in the main text as representative skeletal muscle and cartilage, respectively. Tissues were microdissected, washed in PBS, snap-frozen in liquid nitrogen, and stored at −80 °C until use.

### Preparation of solid tissues

#### Solubilization

Approximately 12 mg of tissue was cut from each liver and sternocleidomastoid and solubilized for proteomics; all other tissues were solubilized in their entirety. Tissues were solubilized in 150 μL of 4% sodium dodecyl sulfate (SDS), 100 mM dithiothreitol, and 100 mM tris-HCl (pH 7.5). Each sample was heated at 95 °C for 6 min to lyse cells and denature endogenous proteases. Next, a probe sonicator was applied for 2 min while using a grinding motion to shred the tissue between the tube and probe. Samples were incubated overnight at 4 °C, incubated at room temperature for 30 min, and then centrifuged at 16,000 × *g* for 5 min to separate insoluble cellular debris. Next, 140 μL of the protein-containing supernatant was transferred to a new LoBind Eppendorf tube; for liver, only 30 μL of supernatant was transferred.

#### Filter-aided sample preparation (FASP)

Cell lysates were prepared using the FASP method^49^. Briefly, 8 volumes of 8 M urea was added to each of the protein-containing supernatants and loaded onto an Amicon 0.5 mL 30,000 molecular-weight-cutoff (MWCO) centrifugal ultrafiltration unit (EMD Millipore). Proteins were washed with 8 M urea, reduced, alkylated with iodoacetamide, washed again with 1 M urea, and then digested with Lys-C (Wako) [40:1 (w/w) protein/enzyme ratio] at 37 °C overnight to generate peptides. The resulting peptides were small enough to pass through the MWCO filter and collected after centrifugation. Lys-C was quenched using 10% trifluoroacetic acid (TFA) to a final concentration of 0.5%. Peptides were desalted using C18 Bond Elut OMIX 100-μL pipette tips (Agilent) and eluted with 0.1% TFA in 70% acetonitrile. Peptides were dried in a SpeedVac and reconstituted in 5% acetonitrile and 0.2% formic acid.

### Preparation of whole blood

One μL of whole blood from each mouse was lysed using 4 μL of 8 M urea and 10 mM tris-HCl (pH 7.5). Samples were bath sonicated for 3 min to shear chromatin. Next, 7.5 μL of 50 mM ammonium bicarbonate was added followed by 1.25 μL of 10 mM dithiothreitol. Samples were incubated at room temperature for 1 h to allow for reduction of cysteines, then 1.25 μL of iodoacetamide was added and incubated without light for 40 min to acetylate cysteines. Samples were diluted with 40 μL of 50 mM ammonium bicarbonate prior to digestion with 1 μg of Lys-C. Samples were digested at 37 °C overnight and quenched with 3.25 μL TFA. Peptides were desalted using C18 Bond Elut OMIX 100 μL pipette tips (Agilent) and eluted with 0.1% TFA in 70% acetonitrile. Peptides were dried in a SpeedVac and reconstituted in 5% acetonitrile and 0.2% formic acid.

### Data acquisition

Approximately 1 μg of reconstituted peptides, as estimated by a BCA assay (Pierce), was injected into a Waters nanoAcquity HPLC coupled to an electrospray ionization Orbitrap mass spectrometer (QE-HF; Thermo Fisher Scientific; Xcalibur 4.0 software). Peptides were separated at 60 °C on a 100 μm-inner-diameter column packed with 20 cm of 1.7 μm ethylene-bridged hybrid C18 particles (Waters) and eluted at 0.3 μL/min in 0.2% formic acid with a gradient of increasing acetonitrile over 2.5 h. A full mass scan [300−1500 mass/charge ratio (m/z)] was performed in the Orbitrap at 60,000 resolution. The 10 most intense MS1 peaks were selected for fragmentation by higher-energy collisional dissociation at 42% collision energy, and the resulting fragments were analyzed with a resolution of 7500 and an isolation width of 2.5 m/z. Dynamic exclusion was enabled with a repeat count of 2 over 30 s and an exclusion duration of 120 s.

### Identification and quantification of peptides and proteins

A customized fork of MetaMorpheus (v. 0.0.305) and mzLib (v. 1.0.470) was used for mass calibration, global post-translational modification discovery (G-PTM-D)^50^, and the identification and quantification of peptides and proteins from the mass spectrometry data. This customized fork allowed for the creation of an averagine^51^ model with a modified ^13^C abundance of 1.2% for peptides containing at least one unlabeled lysine and 2.0% for peptides without an unlabeled lysine, in contrast to the original ^13^C abundance of 1.1%. The source code, executable, and task settings are freely available here: 10.5281/zenodo.5563101^46^. Note that this software is suitable for analysis of data containing peptide mixtures of heavy and light stable-isotope amino acids, commonly referred to as SILAC (Stable Isotope Labeling by Amino acids in Cell culture) whether performed in cells or in larger organisms. All files were searched against a UniProtKB/Swiss-Prot.xml protein database of *Mus musculus* accessed May 19, 2020 with fixed carbamidomethylation of cysteine and variable oxidation of methionine. All other parameters were maintained at their default values except for the addition of variable Lys8 for mass calibration and G-PTM-D, the specification of the SILAC turnover label for quantification, and the allowance of up to one missed proteolytic cleavage. The specification of the SILAC turnover label allowed for the identification and quantification of partially labeled peptides containing a missed cleavage and both a labeled and unlabeled lysine. The intensities for these partially labeled peptides are distributed between the fully labeled and fully unlabeled intensities in the output.

### ApplE Turnover software

We developed an **Application for Elucidating Protein Turnover** (ApplE Turnover) from time-course proteomic data (https://github.com/smith-chem-wisc/AppleTurnover/releases/tag/1.01)^34^. ApplE Turnover accepts MetaMorpheus’s “AllQuantifiedPeptides.psmtsv” output, which contains light and heavy intensities for each peptide found at a 1% FDR in each sample. Timepoints are automatically determined by the program for each MS file if the file contains “_dX_” or “_Xd_” in the file name, where ‘X’ is the timepoint for that file (e.g., “180628_Liver_**3**d_bio1.raw” is interpreted as a day 3 timepoint). The heavy and light intensities are used to create a relative abundance of unlabeled (new) peptide intensity divided by the sum of unlabeled and labeled peptide intensity, ranging from 0 to 1.

ApplE Turnover can readily analyze data from traditional pulse experiments (in which a heavy isotope is administered to unlabeled mice at time zero) as well as our experimental design (in which heavy-labeled mice are administered unlabeled food at time zero). In the current experiment, all unlabeled peptides are newly synthesized because there was no unlabeled lysine available before the unlabeled pulse. In contrast, labeled peptides represent a combination of new and old synthesis because labeled lysine continued to circulate within the mouse after the introduction of unlabeled lysine. For this analysis, relative abundances that had missing values for either the unlabeled or labeled peptide were discarded. Peptides were required to have at least six total valid relative abundances, of which at least three were required from a single timepoint. Thus, each peptide half-life value in this resource results from between 6 and 22 biologically independent replicates (mice). Peptides failing to meet these criteria were discarded, accounting for much of the difference between the numbers of identified and quantified peptides and proteins in this study (Supplementary Fig. 2 and Supplementary Table 1).

Peptide sequences identified by MetaMorpheus are queried against the original protein database to find every possible protein that each sequence could have originated from, effectively undoing protein parsimony. This step prevents peptides that originate from multiple proteins from skewing any of the protein half-lives. Such peptides are reassigned a new protein identifier, where the possible protein accessions are sorted alphabetically and concatenated with ‘|’ as a delimiter. For example, a peptide originating from protein B and protein A would have the distinct protein identifier A | B but would not be considered when determining the half-life for either protein A or B.

**Fig. 8:**
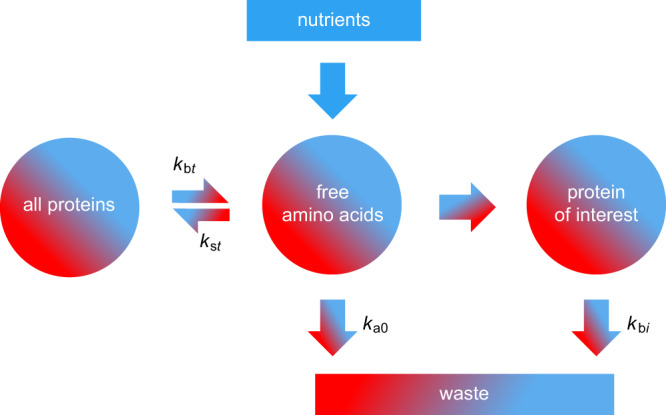
The three-compartment model used by ApplE Turnover. The three compartments (circles) each contain a time-dependent ratio of unlabeled (blue) to labeled (red) lysine. Unlabeled lysine enters the free amino acids compartment from metabolized food. Protein degradation from the all proteins compartment contributes labeled, as well as some unlabeled, lysine to the free amino acids compartment—amino acid recycling from any individual protein of interest is considered negligible. The pool of free amino acids is available for synthesis of all proteins, as well as the protein of interest. Finally, lysine is removed from the system as waste to accommodate nutrient influx and maintain a consistent pool of free amino acids. Note that while this schematic depicts the introduction of unlabeled lysine to a system containing labeled lysine (consistent with the experiments used to generate this resource), the three-compartment model and ApplE Turnover are equally applicable to experiments in which labeled lysine is introduced to a system containing unlabeled lysine.

ApplE Turnover employs a three-compartment model to accurately account for the recycling of heavy lysine in each tissue (Fig. 8). Our implementation of this model utilizes equations 23–28 and the four fitting parameters (*k*_s*t*_, *k*_b*t*_, *k*_a0_, and *k*_b*i*_) reported by Guan and coworkers^23^. We then optimized the computational steps to train the model and fit the data, as explained below.

Although intended for homogenous tissue, this method should be reasonably effective for heterogeneous tissues with multiple cell types because the aggregate recycling rate of all cell types in each tissue is determined from the observed data, which itself arises from all cell types in the tissue. Non-linear regression is used to fit the model to the observed data using the mean squared error (MSE) between the fit and those data as the loss function. Several steps are taken to prevent the model from becoming trapped in a local minimum. First, all peptides are modeled using a set of default parameters and their approximate half-lives are determined. Peptides are then sorted by these provisional half-lives and peptides within the inner quartile range are selected for training. This step helps to prevent training on peptides that originate from sources other than the tissue of interest, such as short half-life blood proteins and poorly-behaved long-lived extracellular matrix proteins. These inner-quartile peptides are sorted by the number of valid values and the sum of all labeled and unlabeled intensities. The top 100 peptides are then used as the initial training set to fit the model. If there are fewer than 100 peptides available, then all peptides are used for training.

A new model is fit to each training peptide by moving each of the three tissue-specific coefficients (*k*_s*t*_, *k*_b*t*_, and *k*_a0_) individually in small steps until the MSE of the fit no longer decreases. At each step, a coefficient representing the peptide half-life (*k*_b*i*_) is also shifted until the MSE no longer decreases for that specific *k*_s*t*_, *k*_b*t*_, or *k*_a0_ step. We discovered that *k*_b*i*_ must be optimized at each step or else a sawtooth behavior occurs, which prematurely stops the nonlinear regression in an artificial local minimum. Once each training peptide has its own set of optimized coefficients, the median value for each tissue-specific coefficient (*k*_s*t*_, *k*_b*t*_, and *k*_a0_) across the 100 training peptides is saved and used as the starting value for a second round of fitting. In this second round, all 100 training peptides are fit together instead of separately, such that the MSE is the average of all peptide fits. The *k*_b*i*_ remains unique to each peptide, but the *k*_s*t*_, *k*_b*t*_, and *k*_a0_ coefficients are forced to be consistent across all peptides. The resulting coefficients are then used as the starting values for a third round of training, this time using the complete set of peptides with half-lives within the inner quartile range.

After a consensus set of tissue-specific coefficients has been established, the user may choose to discard messy peptides [that contain any relative abundance ratio with an abnormally high residual (>0.1)] from the fit. Such messy peptides were discarded for the current analysis. These peptides are typically contaminants, have low intensity, or are false-positive identifications. After the removal of messy peptides, the model is fit a final time using the set of remaining inner-quartile peptides. Next, a grid analysis is implemented as follows to check that the coefficients yield a global, rather than a local, minimum. Each coefficient is modified by a factor of 0.1, 0.2, 0.33, 0.5, 0.9, 1.0, 1.1, 1.25, 2, and 4. There are 12 modification factors and three coefficients, requiring 1728 analyses. For each set of coefficients, the *k*_b*i*_ is optimized for each peptide and the MSE is recorded. If the MSE of the modified set is lower than the MSE of the original set, then the modified coefficients are optimized to reduce MSE and additional rounds of shotgun analysis are used to check for a global minimum.

The global coefficients determined from the training set are then applied to every peptide in the tissue under analysis to determine their respective half-lives. Each peptide half-life value is obtained by a nonlinear regression fit of its 6–22 biologically independent observations to the model (e.g., the orange curve in Fig. 2a); the MSE of this model fit is then calculated. Confidence intervals for these peptide half-lives are generated using a hybrid of the Monte-Carlo and bootstrapping methods. First, the sample standard deviation of the model error for each timepoint of each peptide is calculated using Eq. (1):

1 $$s=\sqrt{\frac{1}{N-1}\mathop{\sum }\limits_{i=1}^{N}{\left({x}_{i}-\bar{x}\right)}^{2}}$$

where *s* is the standard deviation of a single timepoint, *N* is the number of ratios observed for the timepoint, ${x}_{i}$ is the observed ratio, and $\bar{x}$ is the expected ratio for that timepoint. If only one ratio exists for a given timepoint, then the standard deviation is substituted by the absolute difference between the fit and the observed ratio. In the current analysis, we used 200 simulations to model the half-life error for each peptide. For each simulation, the observed ratios are sampled with replacement until the number of simulated ratios for each timepoint matches the original data. These sampled ratios are then modified using the inverse of a normal distribution in combination with a random number generator and the sample standard deviation for each simulated ratio. Finally, these simulated data are are fit using the tissue-specific coefficients to determine the simulated half-lives. This process produces 200 simulated half-lives; the 95% confidence interval is identified between the 5th-shortest and 5th-longest simulated half-lives.

Protein half-lives and confidence intervals (e.g., the orange and red/green curves in Fig. 2c, respectively) are determined using the simulated half-lives of their constituent peptides. All 200 simulations from each peptide of a given protein are pooled, and the median half-life of these simulations is reported as the half-life of the corresponding protein. The 95% confidence interval is the range between the 2.5 and 97.5 percentiles of the simulations.

ApplE Turnover additionally compares between tissues when multiple input files (tissues) are provided (e.g., Fig. 4b). Proteins identified across multiple tissues are selected and a log_2_ fold change and −log_10_(*P*-value) are reported for each shared protein. The *P*-value is calculated by normalizing the observed ratios for all timepoints and implementing a two-tailed *t*-test. Normalization is achieved by finding the average *k*_b*i*_ for the protein in both tissues and creating a normalization timepoint, which is a theoretical timepoint at which both tissues have relative fractions close to 0.5. Ratios are then normalized by subtracting the theoretical ratio at the current timepoint and adding the theoretical ratio at the normalization timepoint. This process maintains the standard deviation of the ratios and allows for a consistent comparison across tissues. In addition to comparing protein turnover rates across tissues, this method is also used to determine differences in proteoform half-lives caused by PTMs. For this resource, we considered these proteoform half-life differences significant if they had a log_2_ fold change > 0.25 and a *P*-value < 0.0001. Comparisons involving oxidation of methionine and/or proteins with half-lives < 3 d were omitted.

Despite the multiple training rounds, numerous analyses to check for a global minimum, and calculation of confidence intervals, the processing of our data in ApplE Turnover required only three minutes per tissue on a quad-core computer.

### Reporting summary

Further information on research design is available in the Nature Research Reporting Summary linked to this article.

## Supplementary information

Supplementary Information

Description of Additional Supplementary Files

Supplementary Data 1

Supplementary Data 2

Supplementary Data 3

Reporting Summary

## Data Availability

All acquired and analyzed data, as well as the xml search database downloaded from UniProt, have been deposited in the MassIVE repository (https://massive.ucsd.edu/) under the dataset identifier MSV000086426 and are available at 10.25345/C5RB7F. A tutorial for users has been provided (Supplementary Note 1). Source data are provided with this paper.

## Source data

Source Data

## Code availability

The source code and executable for the customized MetaMorpheus (https://github.com/smith-chem-wisc/MetaMorpheus) and mzLib (https://github.com/smith-chem-wisc/mzLib) branches used in this resource are available at 10.5281/zenodo.5563101^46^. The source code and executable for ApplE Turnover are available at https://github.com/smith-chem-wisc/AppleTurnover/releases/tag/1.01 or from 10.5281/zenodo.5555092^34^. The ApplE Turnover executable file is also available in the MassIVE repository (10.25345/C5RB7F) with the data.
