# Supplementary material for: An atlas of protein turnover rates in mouse tissues
Source: Nat Commun. 2021 Nov 26;12:6778. doi: 10.1038/s41467-021-26842-3 (PMC8626426; doi:10.1038/s41467-021-26842-3)
Supplement: Supplementary file 1 — Supplementary Information [file 41467_2021_26842_MOESM1_ESM.pdf]

Supplementary Information

for

An Atlas of Protein Turnover Rates in Mouse Tissues

TABLE OF CONTENTS

Supplementary Figures

1. Results of control experiment 2
2. Relationship between peptide intensity and quantification 3
3. Comparison of the three analyzed cartilages 4
4. Comparison of the two analyzed skeletal muscles 5
5. Example of a blood protein in skeletal muscle 6
6. Relationship between calculated protein half-lives and their 95% confidence intervals. 7
7. Comparison of peptide half-lives from ApplE Turnover and Turnover GUI 8
8. Validation analysis using peptides containing a single missed cleavage 9

Supplementary Tables

1. Number of proteins and peptides identified and quantified across all tissues 11
2. Agreement between multiple peptides for each protein 12

Supplementary Note 1—Tutorial

1. Access the Data *(Download half-lives as .tsv)* 13
2. Visualize the Data *(Download and use ApplE Turnover)* 17
3. Analyze Data with ApplE Turnover *(Analyze new tissues)* 22

SUPPLEMENTARY FIGURES

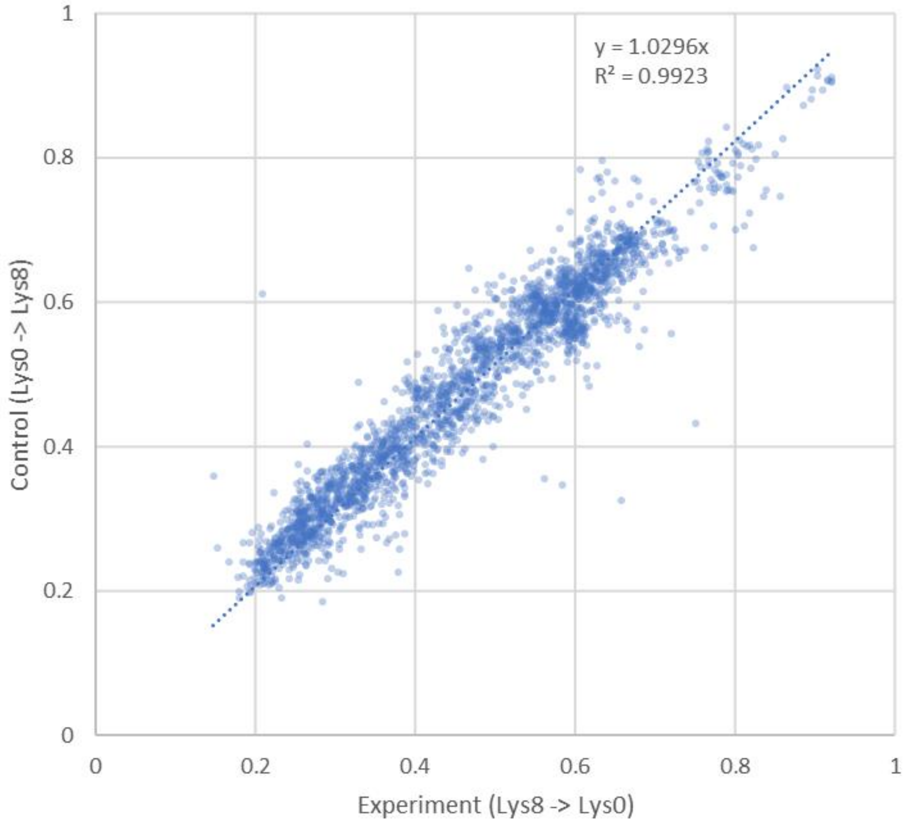

Supplementary Figure 1. Results of the control experiment used to validate the experimental design where ~99% heavy-labeled (Lys8) NBSGW mice were switched to unlabeled (Lys0) food at day zero. Protein turnover experiments typically do the opposite and switch unlabeled mice to a heavy-labeled diet at day zero. To confirm that this difference does not affect our protein half-life measurements, we performed a control experiment that uses the traditional labeling direction and a more common mouse strain (C57/BL6J). Briefly, tissues from control mice (Online Methods—Mice) were harvested at 14 d after introduction of Lys8 and compared with tissues of 14 d mice from the resource. The relative fractions for the experiment (x-axis) and the control (y-axis) are plotted, and each datapoint represents the average ratio across all 14 d replicates for a single peptide in cartilage. The excellent correlation between these relative fraction values, the only raw data used to calculate protein half-lives, demonstrates that neither the directionality of the labeling nor the mouse strain influences the protein turnover rates reported in this resource. Source data are provided with this paper; raw mass spectrometry data are available as described in the Data Availability section.

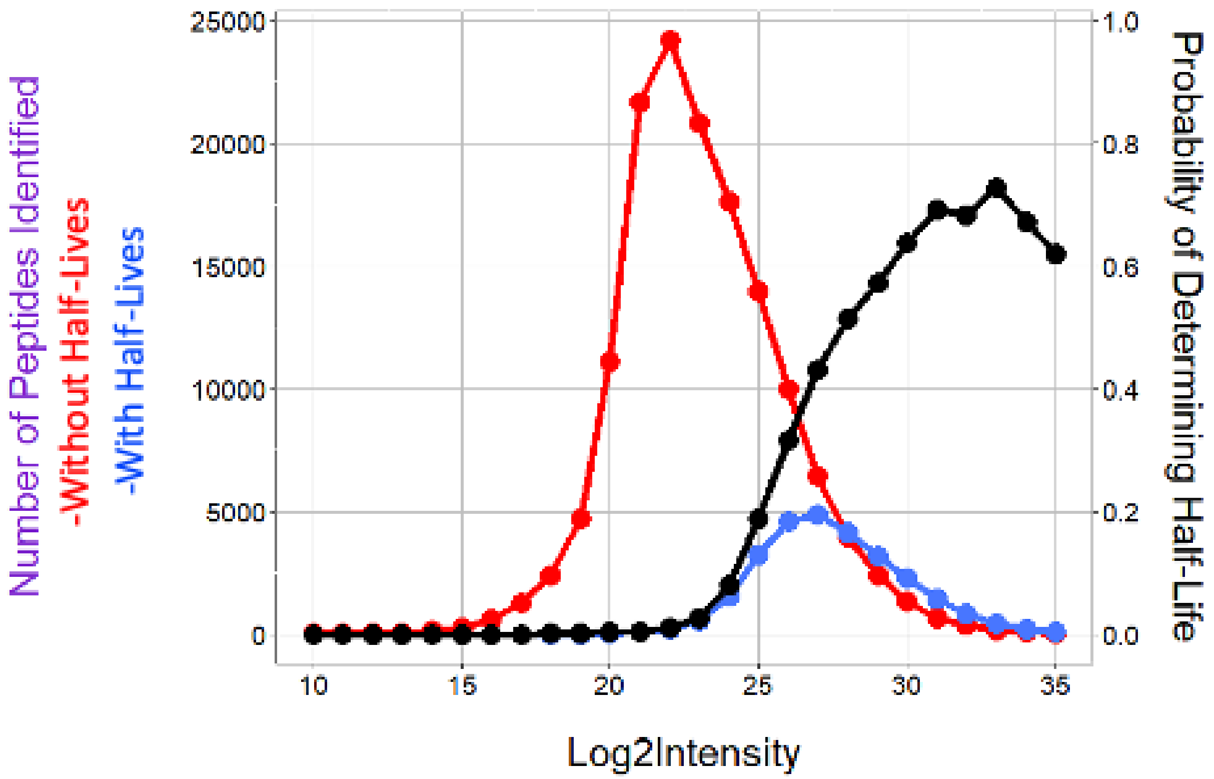

Supplementary Figure 2. Proteins and peptides that are observed at higher intensities are more likely to yield half-life measurements than lower intensity species. All peptides from all tissues were aggregated for this analysis. The most intense biological replicate was used to represent the intensity of each peptide. Only 16% of peptides were quantified; however, 57% of identified proteins were quantified (Table S1) because many of the lower intensity peptides are not necessary for quantification of a protein as long as at least one of its peptides has a relatively high intensity. Source data are provided with this paper; raw mass spectrometry data are available as described in the Data Availability section.

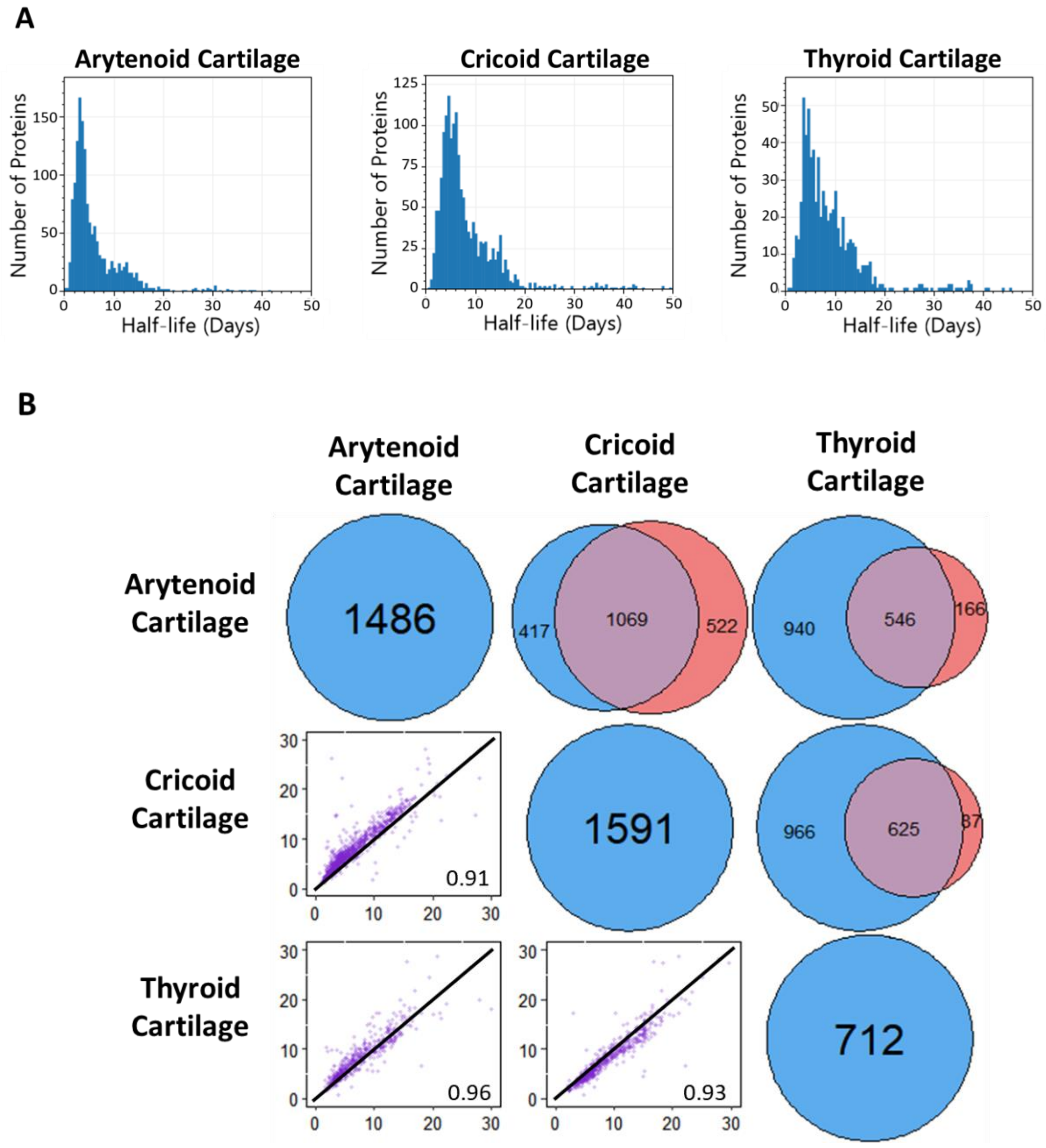

Supplementary Figure 3. Comparison of protein turnover rates across the three analyzed cartilages (cricoid, arytenoid, and thyroid). A) Half-life distributions for the three cartilages, and B) similarity of protein half-lives in pairwise comparisons of the three tissues. Source data are provided with this paper; raw mass spectrometry data are available as described in the Data Availability section.

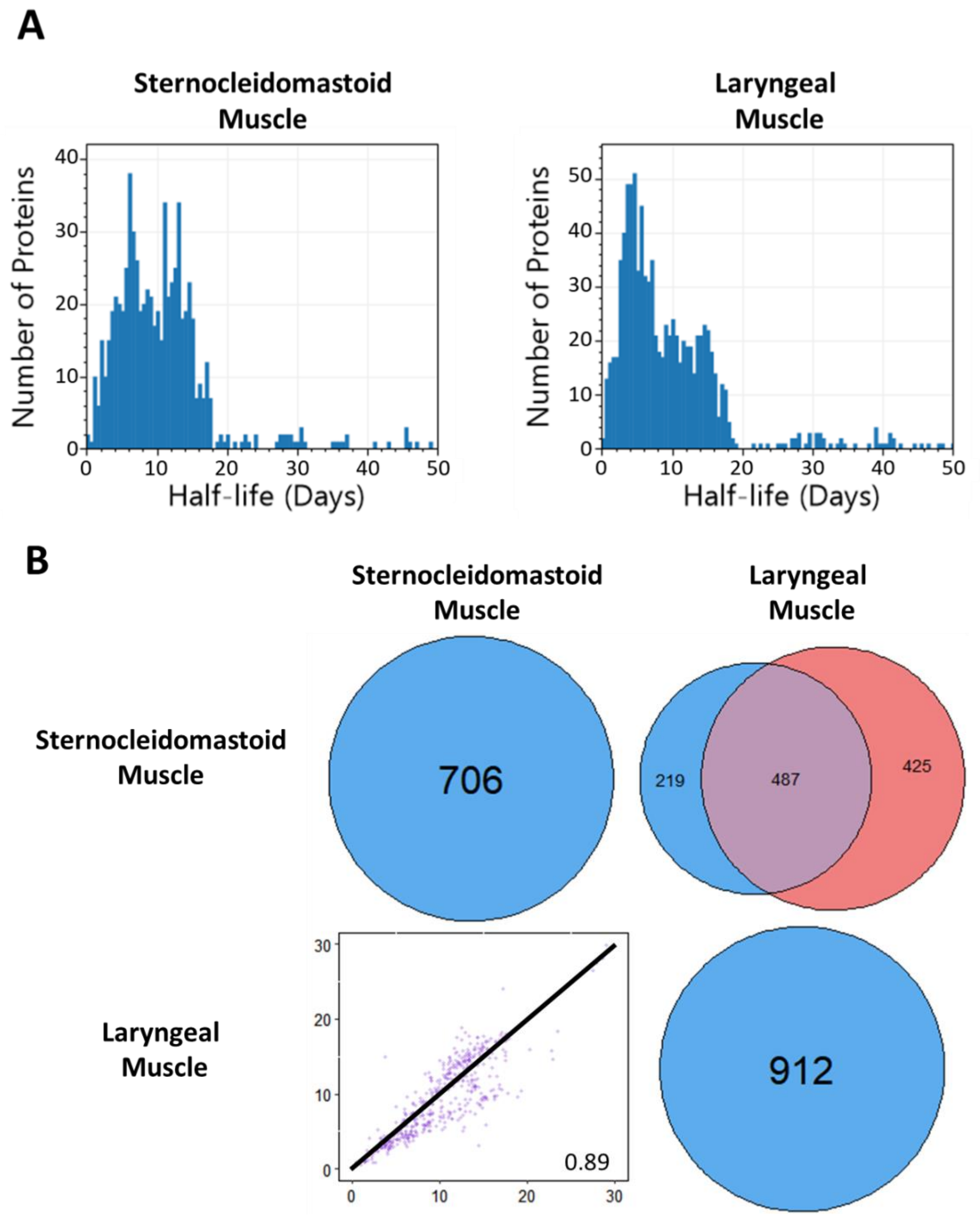

Supplementary Figure 4. Comparison of protein turnover rates between the two skeletal muscles (sternocleidomastoid and intrinsic laryngeal). A) Half-life distributions for the muscles and B) similarity of protein half-lives. Source data are provided with this paper; raw mass spectrometry data are available as described in the Data Availability section.

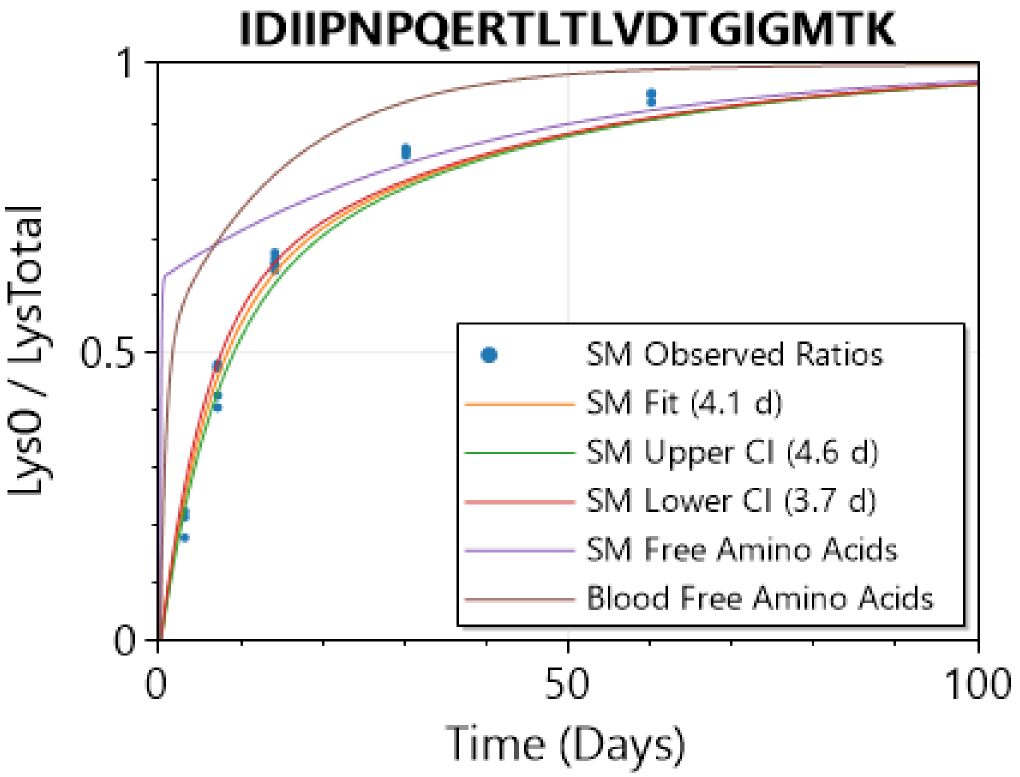

Supplementary Figure 5. Plot showing turnover data in skeletal muscle (SM) for a peptide from albumin. It should not be possible for any blue data points to be above the purple curve, which represents the turnover for free amino acids in skeletal muscle. However, this occurs because albumin is not synthesized in the muscle tissue itself using that pool of amino acids. Notice that the data points do fall below the brown curve, which represents free amino acid turnover in blood. Source data are provided with this paper; raw mass spectrometry data are available as described in the Data Availability section.

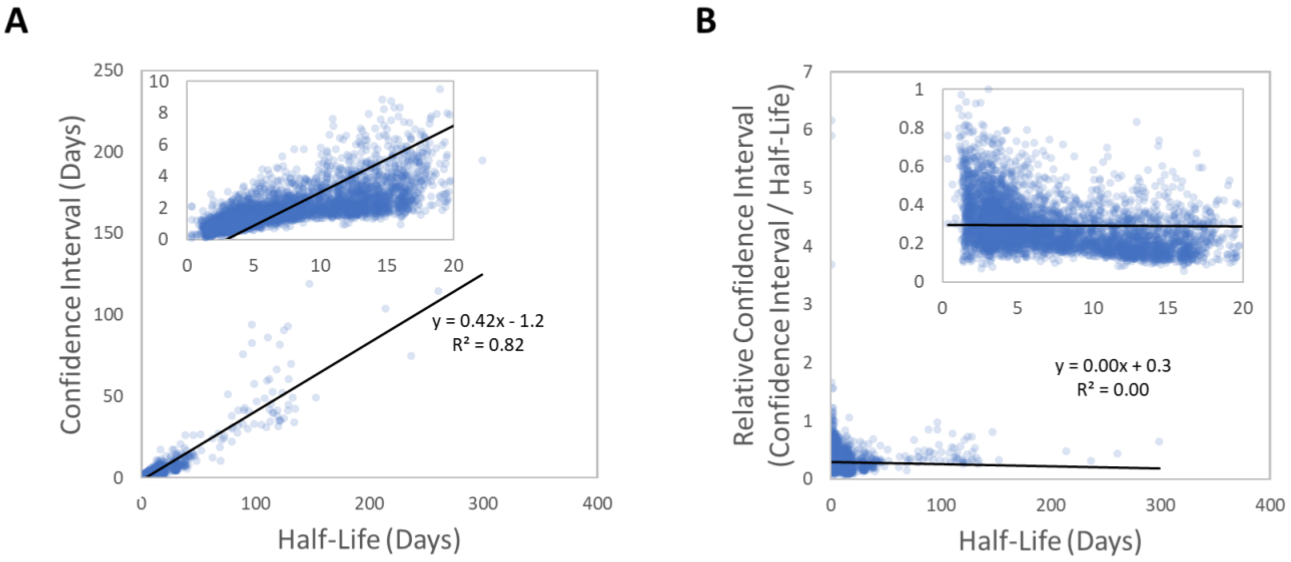

Supplementary **Figure 6. Relationship between calculated protein half-lives and their 95% confidence intervals**. (**A**) The confidence interval of a protein half-life is positively correlated with the half-life itself, such that (**B**) the relative confidence interval is independent of the half-life value. The average relative confidence interval is 0.30 (median = 0.26) meaning, for example, that a half-life of 10 d will typically have a 95% confidence interval on the order of 8.5-11.5 d. Plot (**B**) additionally shows that long-lived proteins and extremely-short-lived proteins typically have larger confidence intervals than proteins with mid-range half-lives, which is unsurprising given that the experimental timepoints of this study spanned 3 to 60 d and that the three-compartment model is not ideal for long-lived proteins, as described in the Results section of the main text. Source data are provided with this paper; raw mass spectrometry data are available as described in the Data Availability section.

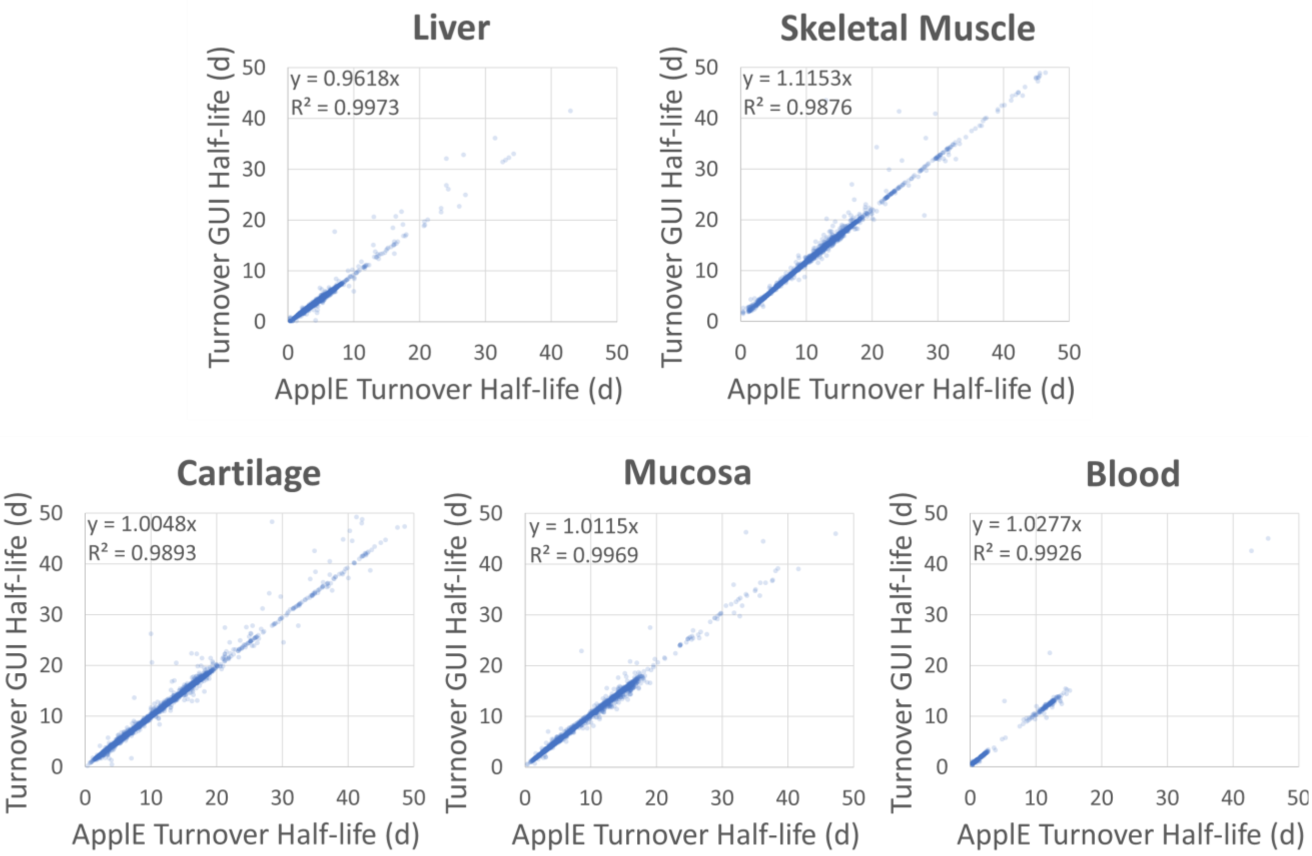

Supplementary Figure 7. Comparison of calculated peptide half-lives from the ApplE Turnover and Turnover GUI software programs. Both programs were provided with the same peptide-level quantification results from MetaMorpheus. The two programs have minor differences in their approaches to nonlinear regression. We observed that Turnover GUI occasionally arrives at different global fitting parameters (a, b, and r) when provided with unique sets of starting parameters. For this comparison, we used the default fitting parameters as starting parameters (a = 0.034277, b = 0.44487, r = 11.8366) and used all peptides with at least 11 valid values for training. Source data are provided with this paper; raw mass spectrometry data are available as described in the Data Availability section.

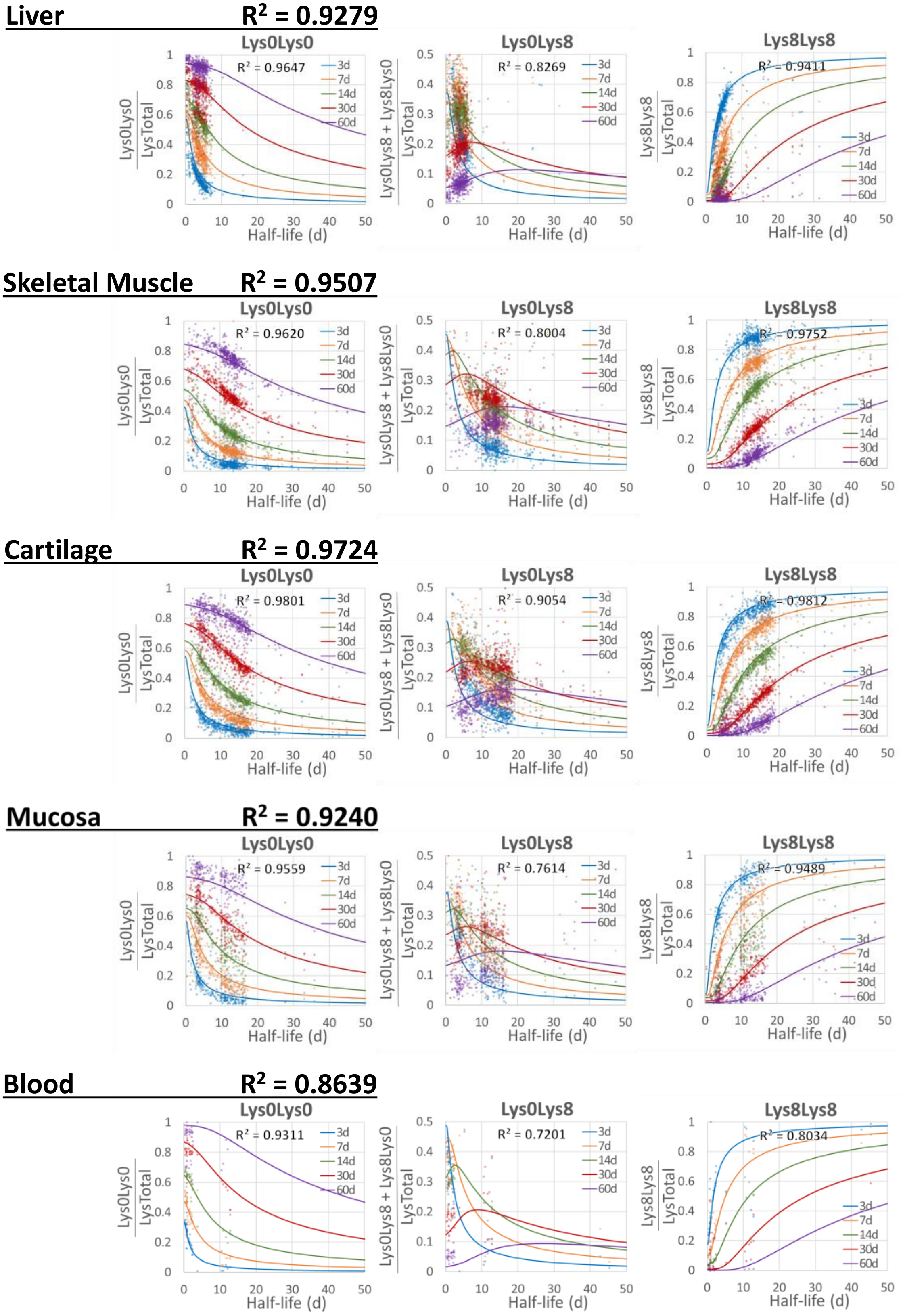

Supplementary Figure 8. Legend on next page . . .

Supplementary Figure 8. Validation analysis for the five tissue classes using peptides containing a single missed cleavage.

Figure Description: These peptides can contain two Lys0 residues (left plots), two Lys8 residues (right plots), or one of each (middle plots)—note that Lys0Lys8 and Lys8Lys0 are grouped together because their abundances are indistinguishable in the LC-MS1 data used for quantification. We used the three-compartment model to predict the relative fraction of these missed cleavage peptides over time and compare these predictions with our observed data. The solid lines are the predicted ratios for each measured timepoint and are a function of the peptide half-life. The points are the observed data, where each point is the average of all biological replicates for a single missed-cleavage peptide at a single timepoint. Source data are provided with this paper; raw mass spectrometry data are available as described in Data Availability.

Discussion of Results: These plots and the correlation coefficients show good agreement between the expected relative abundances and the experimentally observed abundances. Four tissues have R^2 values > 0.92. Blood is somewhat lower at $R^2 = 0.86$, which is not surprising given that the three-compartment model is not well-suited to turnover of blood proteins that are generally not synthesized nor degraded within blood itself, as discussed in the Results section of this paper. For all tissues, the fits are very good for the Lys0Lys0 (left) and Lys8Lys8 (right) plots and less good for the Lys0Lys8 (middle) plots, but even those fractional abundances are consistent with the expected trends. We note that these missed cleavage peptides (used in this validation analysis) have a higher identification false discovery rate (FDR) and less precise quantification values than the peptides without missed cleavages (predominant ones used in calculation of protein turnover rates in this resource). This stems from the missed cleavage peptides typically having lower abundances in the sample (as they require that LysC missed cleaving the protein at that site) and also that the ion intensity is necessarily split among the 3 isotopic versions. Nonetheless, there is good agreement between the observed data and the predicted ratios, providing confidence that the model used by ApplE Turnover is acceptable for determining peptide and protein half-lives.

Additional Detailed Explanation: The three-compartment model was fitted to the data in this resource to determine peptide and protein half-lives from peptide quantification results. A visual representation of this fit can be viewed in Figure 2B, where the expected relative isotope abundance for Lys0 is plotted across a range of half-lives for each measured timepoint. The fit in Figure 2B assumes that each peptide has two states: either it contains a Lys0 or a Lys8. However, the LysC protease (used to digest proteins into peptides during sample preparation) is known to occasionally skip lysine residues, which results in a missed cleavage and yields peptides with more than one lysine residue. A peptide with one missed cleavage can have two Lys0, two Lys8, or both a Lys0 and a Lys8. For the overall turnover analysis in this resource, the Lys0Lys0 peptides and Lys8Lys8 peptides were considered unlabeled and labeled, respectively; but the partially-labeled (Lys0Lys8) peptides required splitting the peak intensities between the fully-unlabeled (Lys0) and fully-labeled (Lys8) categories according to the proportion of Lys0 and Lys8 in the partially labeled peptide(s) (e.g. Lys0Lys8 intensities [1 missed cleavage] are split equally between Lys0 and Lys8, while Lys0Lys0Lys8 [2 missed cleavages] have two-thirds of their observed intensities assigned to Lys0 and one-third to Lys8).

SUPPLEMENTARY TABLES

Supplementary Table 1. Number of proteins and peptides identified and quantified across all tissues in this resource.

Tissue	Identified Peptides	Peptides with Half-lives	Identified Proteins	Proteins with Half-lives	Fraction of Proteins Quantified
**Main Tissues**					
Liver	34879	8374	3794	2004	0.53
Skeletal Muscle *(Sternocleidomastoid)*	16908	3697	1767	706	0.40
Cartilage *(Cricoid)*	33881	5507	3765	1591	0.42
Mucosa *(Vocal Fold)*	21777	2388	2740	753	0.27
Whole Blood	7266	544	633	155	0.24
**Additional Tissues**					
Intrinsic Laryngeal Muscle	26826	3874	2972	912	0.31
Thyroid Cartilage	27251	1560	2948	712	0.24
Arytenoid Cartilage	30211	5357	3539	1486	0.42
**Summary**					
All Tissues *(Total)*	198999	31301	22158	8319	0.38
All Tissues *(Unique)*	76687	15591	5413	3106	0.57

Supplementary Table 2. Agreement between multiple peptides for each protein. (Note: Confidence interval is denoted C.I. in the column headings.) ApplE Turnover calculates peptide half-lives prior to compiling them into protein half-lives, which allows a built-in validation by comparing the multiple peptide half-lives determined for each protein. The results in this Table show an average of four peptide half-life values went into each protein half-life, 99% of peptide half-lives fall within the confidence intervals of their protein's half-life, 90% of peptide confidence intervals overlap those of the other peptides for that protein, and 73% of peptide half-lives fall within the confidence intervals of the other peptides for that protein. This agreement between peptides is not simply a result of wide confidence interval ranges, as the median is only 1.4d (i.e. half-life ±0.7d). The consistency of these independently determined half-lives for multiple peptides from the same protein demonstrate the good precision of the protein turnover rates reported in this resource.

Additional Detailed Explanation: Not all peptides from a protein will have identical turnover rates, as certain peptides arise from different proteoforms (e.g. *in vivo* truncation or PTM. Shorter proteoforms than the intact protein can be generated through alternative translation start codons or post-translational processing and have been shown to cause differences in proteoform stability (https://www.embopress.org/doi/full/10.15252/msb.20156662). This discrepancy in peptide half-life measurements underscores the importance for measuring protein turnover rates on the peptide level rather than the protein level and highlights the capabilities of ApplE Turnover to visualize individual peptide measurements and find proteoform-specific differences in turnover rates.

Tissue	Average Number of Peptides per Protein	Fraction of Proteins with Multiple Peptides	Fraction of Peptide Half-lives within the C.I. of its Protein	Fraction of Peptide C.I.s Overlapping Other Peptide C.I.s	Fraction of Peptide Half-lives within Other Peptide C.I.s
**Liver**	4.18	0.61	0.998	0.96	0.84
**Skeletal Muscle**	5.24	0.52	0.990	0.76	0.55
**Cartilage**	3.46	0.50	0.995	0.85	0.65
**Mucosa**	3.17	0.49	0.998	0.94	0.74
**Blood**	3.51	0.46	0.998	0.99	0.88
**AVERAGE**	3.91	0.52	0.996	0.90	0.73

SUPPLEMENTARY NOTE 1 — TUTORIAL

1. ACCESS THE DATA

This resource is hosted through MassIVE with the identifier “MSV000086426”. Below is a quick guide for downloading these data through ftp.

1.1. Download and install an ftp client. We use FileZilla: https://filezilla-project.org/download.php?type=client

1.2. Open FileZilla.

1.3. In the top bar, enter the following information:
If you are a reviewer, the dataset is still private and can be securely viewed with the following fields:

Host: ftp://MSV000086426@massive.ucsd.edu

Username: MSV000086426_reviewer

Password: ApplETurnover

Port: 21

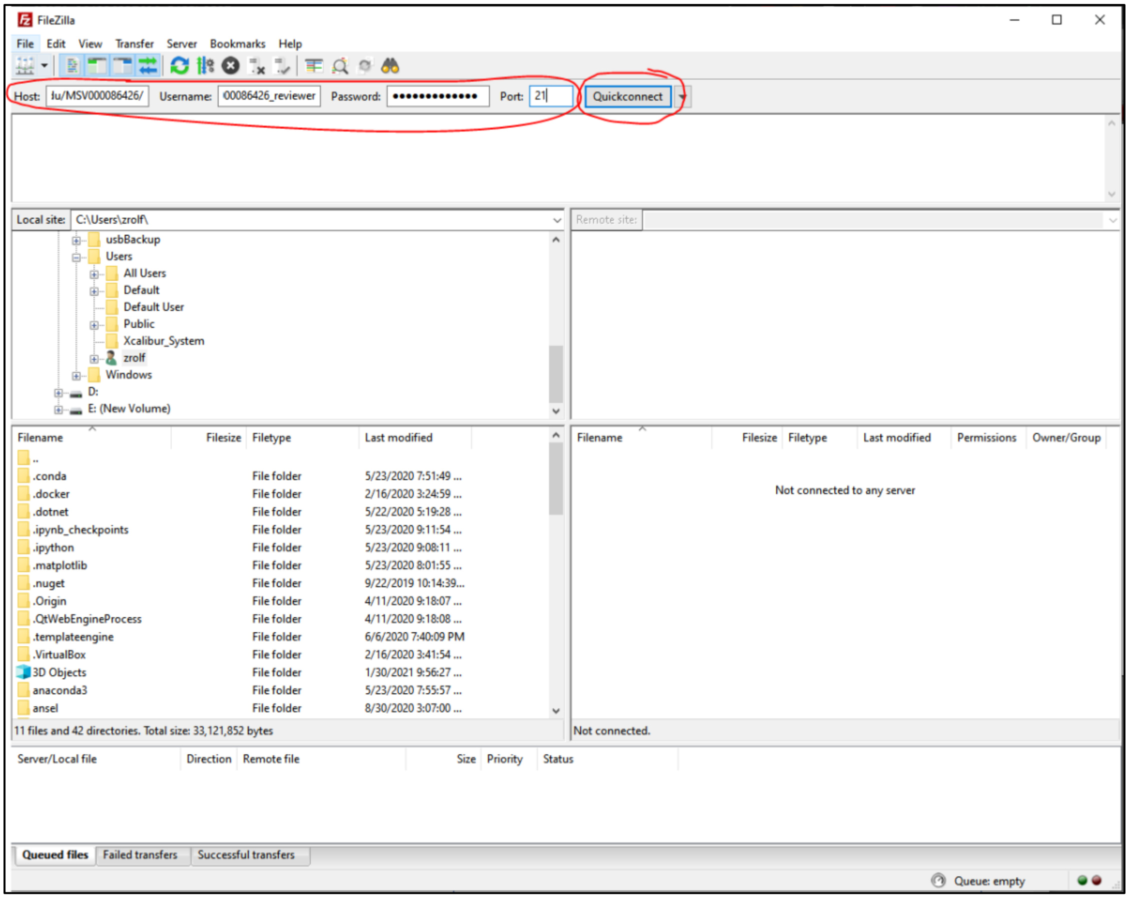

If you are not a reviewer, the dataset is now public and can be found with the following fields:

Host: ftp://massive.ucsd.edu/MSV000086426/

Username: {leave blank}

Password: {leave blank}

Port: 21

1.4. Click "Quickconnect". (You may get a message about an insecure FTP connection, but it should be OK to proceed.)

1.5. In Right Panel "Remote site", navigate the folders:
updates -> 2021-02-12_zrolfs_27ac70a9 -> other

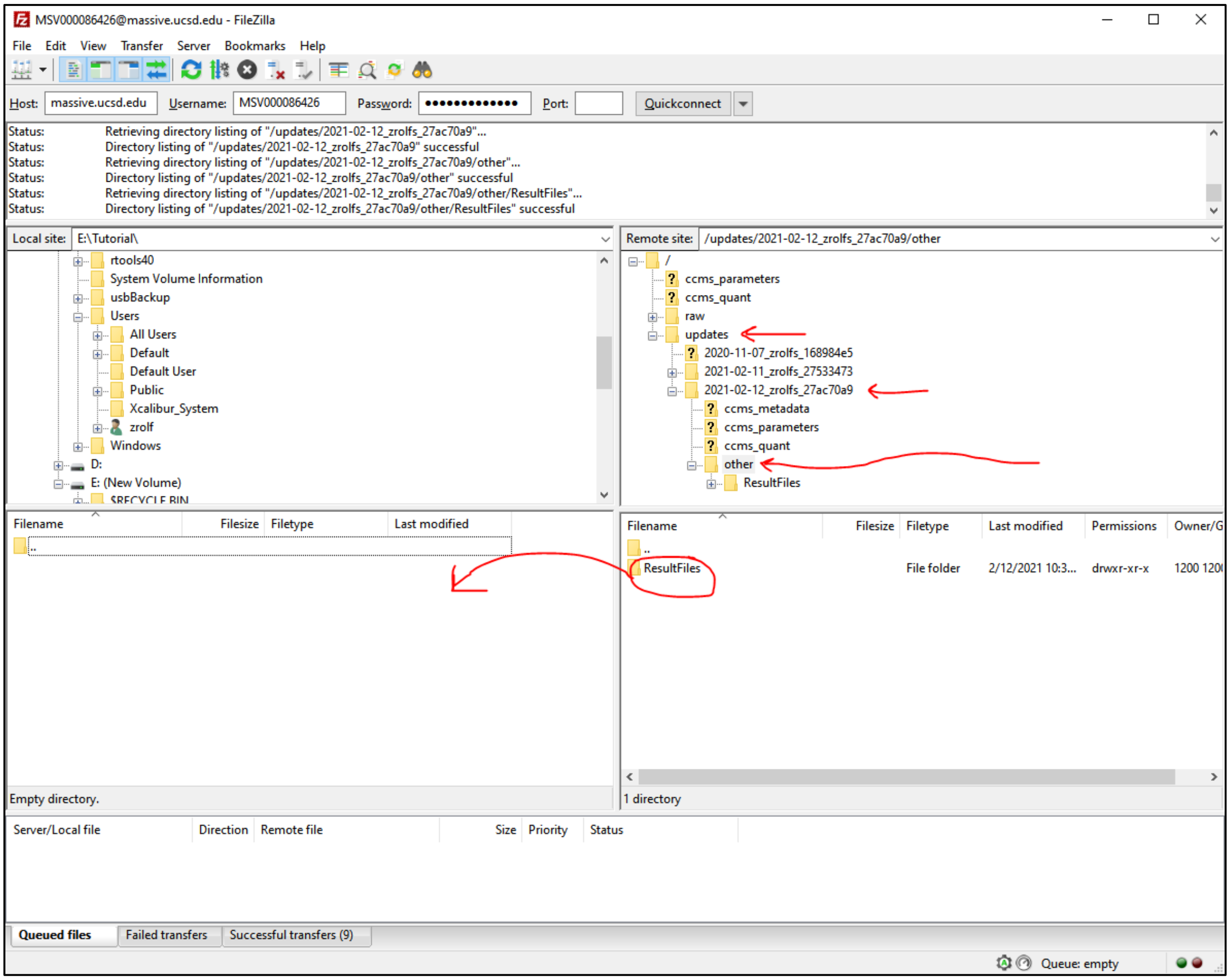

1.6. Drag and drop the "ResultFiles" folder (from the right side) onto your local directory (left side). This folder contains all of the calculated half-lives and statistical comparisons produced by ApplE Turnover.

1.7. Locate and open the "ResultFiles" folder on your machine (not within the FTP Client). It will display a separate folder for each tissue in this resource.

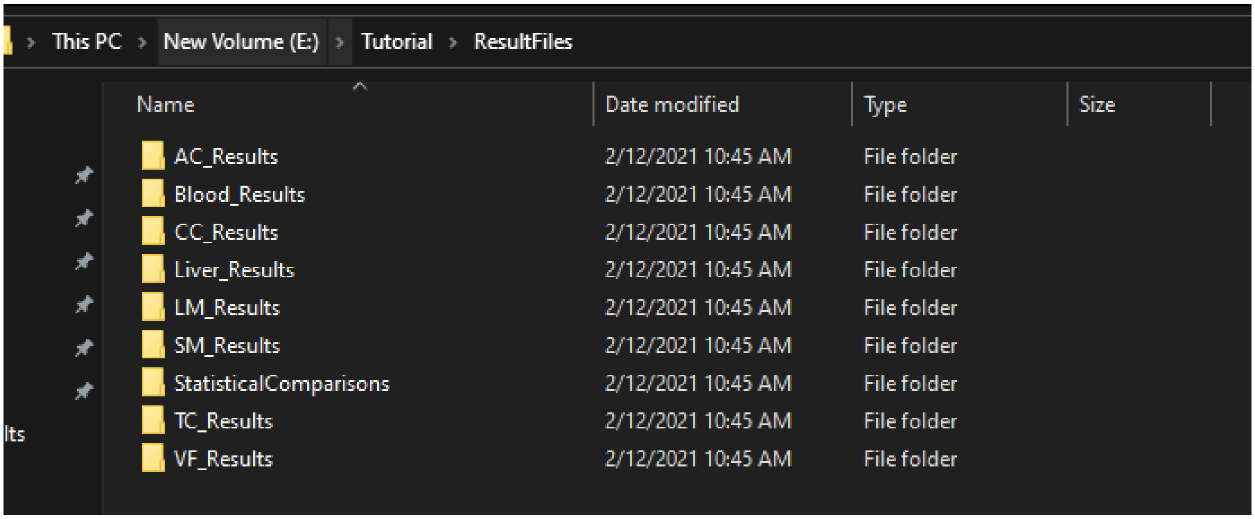

AC_Results: Results for Arytenoid Cartilage
Blood_Results: Results for Blood (Blood in manuscript)
CC_Results: Results for Cricoid Cartilage (Cartilage in manuscript)
Liver_Results: Results for Liver (Liver in manuscript)
LM_Results: Results for Intrinsic Laryngeal Muscle
SM_Results: Results for Sternocleidomastoid Muscle (Skeletal Muscle in manuscript)
StatisticalComparisons: -log10(P-value) and log2(fold change) for protein half-life comparisons across tissues.
TC_Results: Results for Thyroid Cartilage
VF_Results: Results for Vocal Fold Mucosa (Mucosa in manuscript)

1.8. Open the folder for your tissue of interest. Arytenoid Cartilage has been selected as an example.

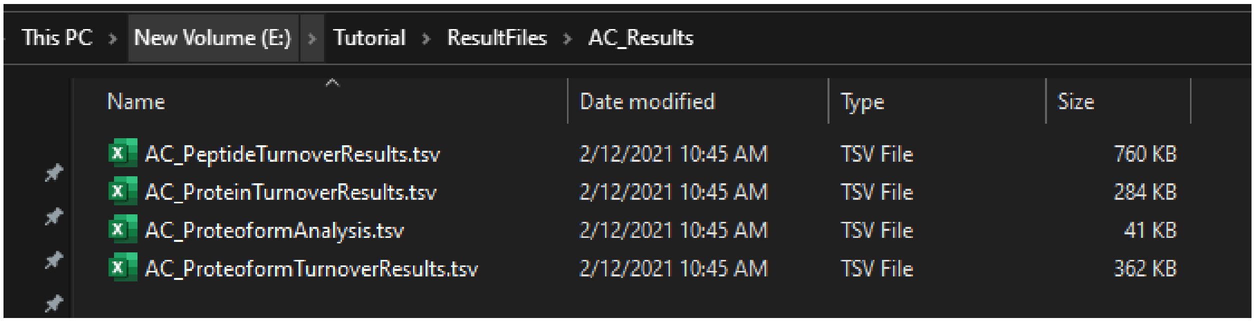

***_PeptideTurnoverResults.tsv**: Half-lives and confidence intervals for all peptides.
***_ProteinTurnoverResults.tsv**: Half-lives and confidence intervals for all proteins.
***_ProteoformAnalysis.tsv**: Comparisons between modified and unmodified proteoforms.
***_ProteoformTurnoverResults.tsv**: Contains half-lives and confidence intervals for all proteoforms.

1.9. Most researchers are interested in the protein half-lives, which can be found in "*_ProteinTurnoverResults.tsv"

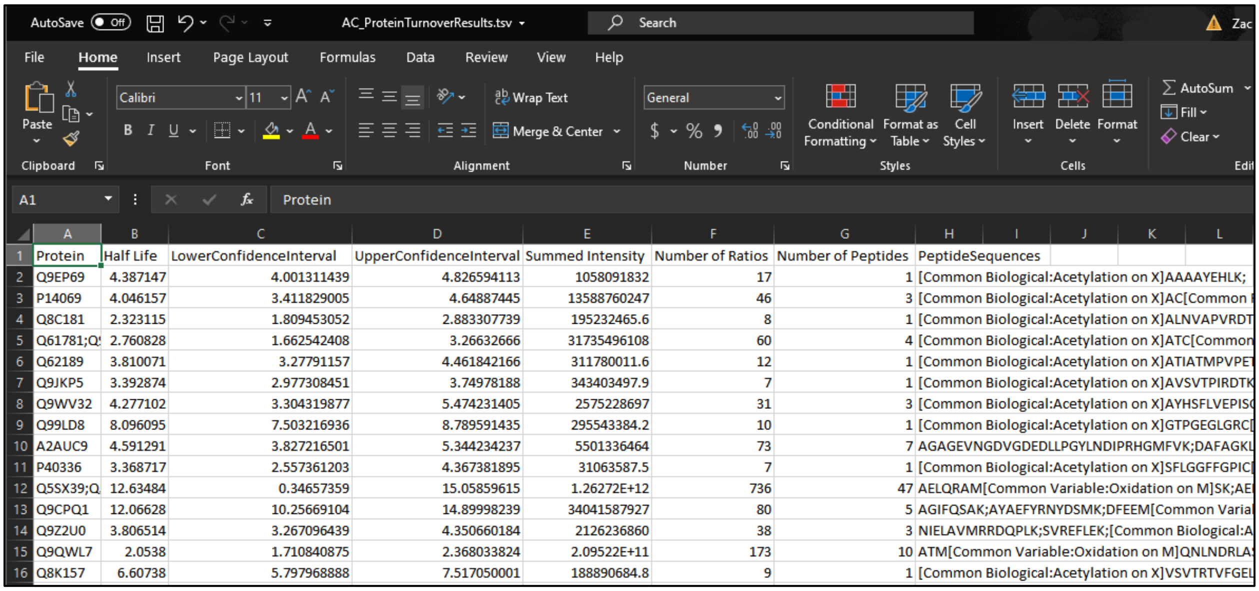

Protein	Half Life	LowerConfidenceInterval	UpperConfidenceInterval	Summed Intensity	Number of Ratios	Number of Peptides	PeptideSequences
Q9EP69	4.387147	4.001311439	4.826594113	1058091832	17	1	[Common Biological:Acetylation on X]AAAAYEHLK;
P14069	4.046157	3.411829005	4.64887445	13588760247	46	3	[Common Biological:Acetylation on X]AC[Common
Q8C181	2.323115	1.809453052	2.883307739	195232465.6	8	1	[Common Biological:Acetylation on X]ALNVAPVRDT
Q61781;Q	2.760828	1.662542408	3.26632666	31735496108	60	4	[Common Biological:Acetylation on X]ATC[Common
Q62189	3.810071	3.27791157	4.461842166	311780011.6	12	1	[Common Biological:Acetylation on X]ATIATMPVPE
Q9JKP5	3.392874	2.977308451	3.74978188	343403497.9	7	1	[Common Biological:Acetylation on X]AVSVTPIRDTK
Q9WV32	4.277102	3.304319877	5.474231405	2575228697	31	3	[Common Biological:Acetylation on X]AYHSFLVEPIS
Q99LD8	8.096095	7.503216936	8.789591435	295543384.2	10	1	[Common Biological:Acetylation on X]GTPGEGLGRC
A2AUC9	4.591291	3.827216501	5.344234237	5501336464	73	7	AGAGEVNGDVGDEDLLPGYLNDIPRHGMFVK;DAFAGKL
P40336	3.368717	2.557361203	4.367381895	31063587.5	7	1	[Common Biological:Acetylation on X]SFLGGFFGPIC
Q5SX39;Q	12.63484	0.34657359	15.05859615	1.26272E+12	736	47	AELQRAM[Common Variable:Oxidation on M]SK;AE
Q9CPQ1	12.06628	10.25669104	14.89998239	34041587927	80	5	AGIFQSAK;AYAEFYRNYDSMK;DFEEM[Common Varia
Q9Z2U0	3.806514	3.267096439	4.350660184	2126236860	38	3	NIELAVMRRDQPLK;SVREFLEK;[Common Biological:A
Q9QWL7	2.0538	1.710840875	2.368033824	2.09522E+11	173	10	ATM[Common Variable:Oxidation on M]QNLNDRLA
Q8K157	6.60738	5.797968888	7.517050001	188890684.8	9	1	[Common Biological:Acetylation on X]VSVTRTVFGEL

Protein: The Uniprot protein accession number

Half Life: The protein half-life (measured in days)

LowerConfidenceInterval: Lower end of the 95% confidence interval (measured in days)

UpperConfidenceInterval: Upper end of the 95% confidence interval (measured in days)

Summed Intensity: Summed intensity of all isotopic envelopes for all peptides from this protein

Number of Ratios: Number of Lys0:Lys8 ratios obtained for this protein

Number of Peptides: Number of peptide half-lives used to determine this protein half-life

PeptideSequences: Peptide sequences that were quantified for this protein. Sequences are separated by ";".

2. VISUALIZE THE DATA

Visualization and data processing with ApplE Turnover requires a Windows operating system.

2.1. Return to the ftp client (e.g. FileZilla).

2.2. Navigate to updates -> 2021-02-11_zrolfs_27533473

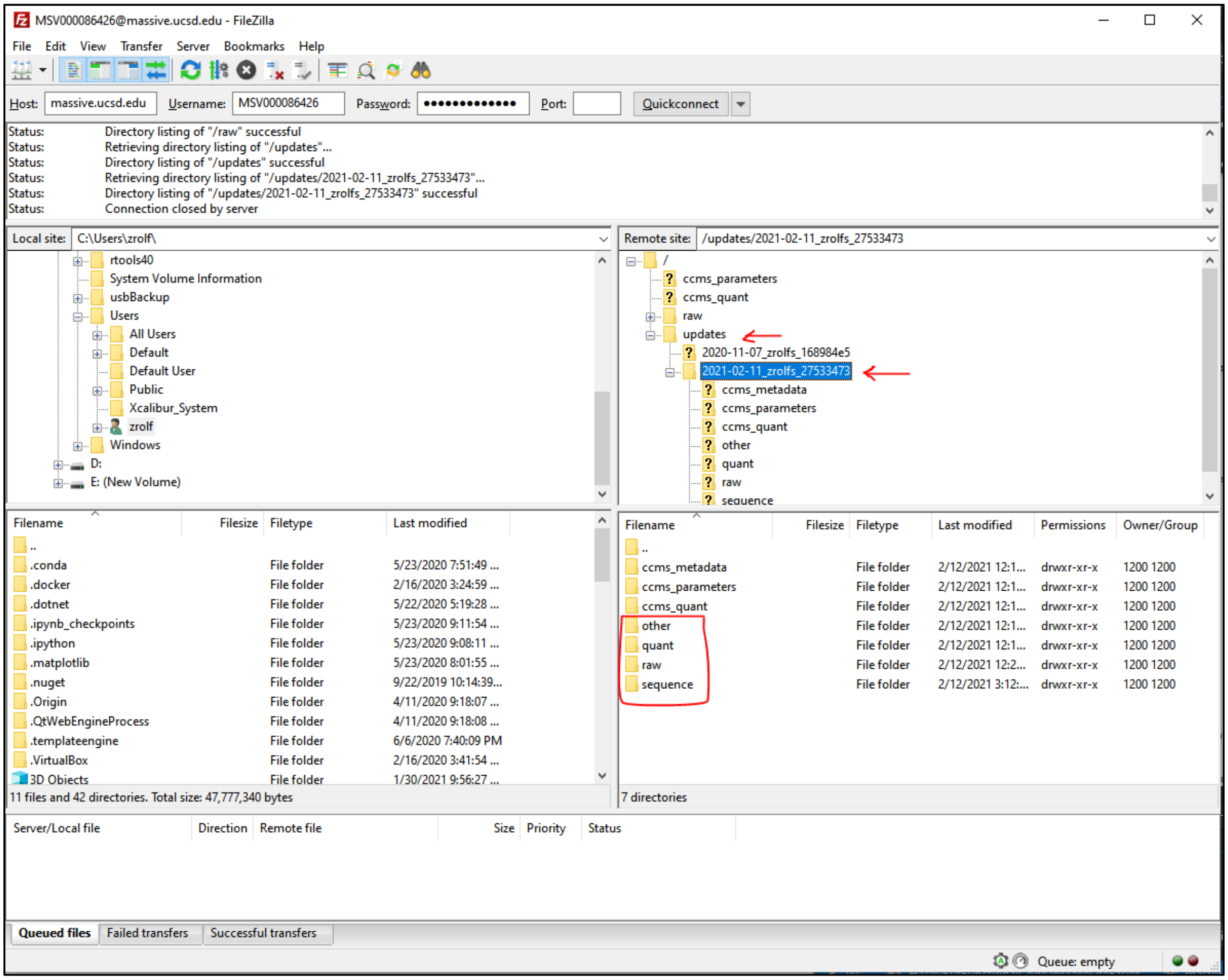

2.3. There are four folders of potential interest here:

-**other**: Contains the ApplE Turnover software and files to quickly visualize the resource.

-**quant**: Contains the quantified intensities of all peptides containing Lys0 and Lys8. These data are used as input for ApplE Turnover.

-**raw**: Contains the raw mass spectrometry data files used in this resource.

-**sequence**: The protein database used during the data analysis. This database is used as input for ApplE Turnover.

Drag and drop the "other" folder (from the right side) onto your local directory (left side).

2.4. Locate the "other" folder in your file explorer.

2.5. Unzip "ApplETurnoverSoftware.zip" (right click and select "Extract All")

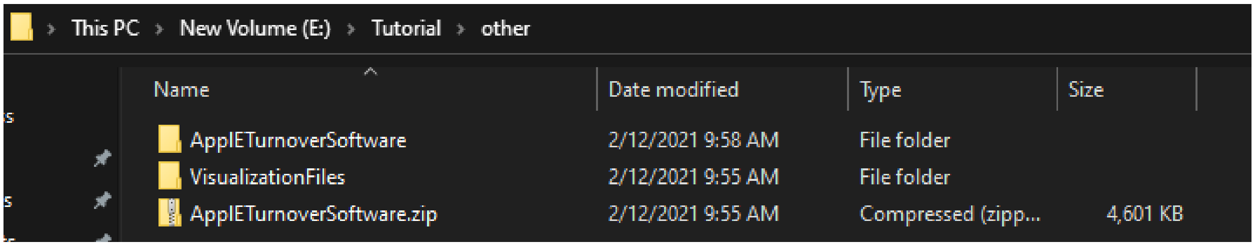

2.6. Open "AppleTurnover.exe", which is located in the unzipped folder "ApplETurnoverSoftware". The following screen should appear.

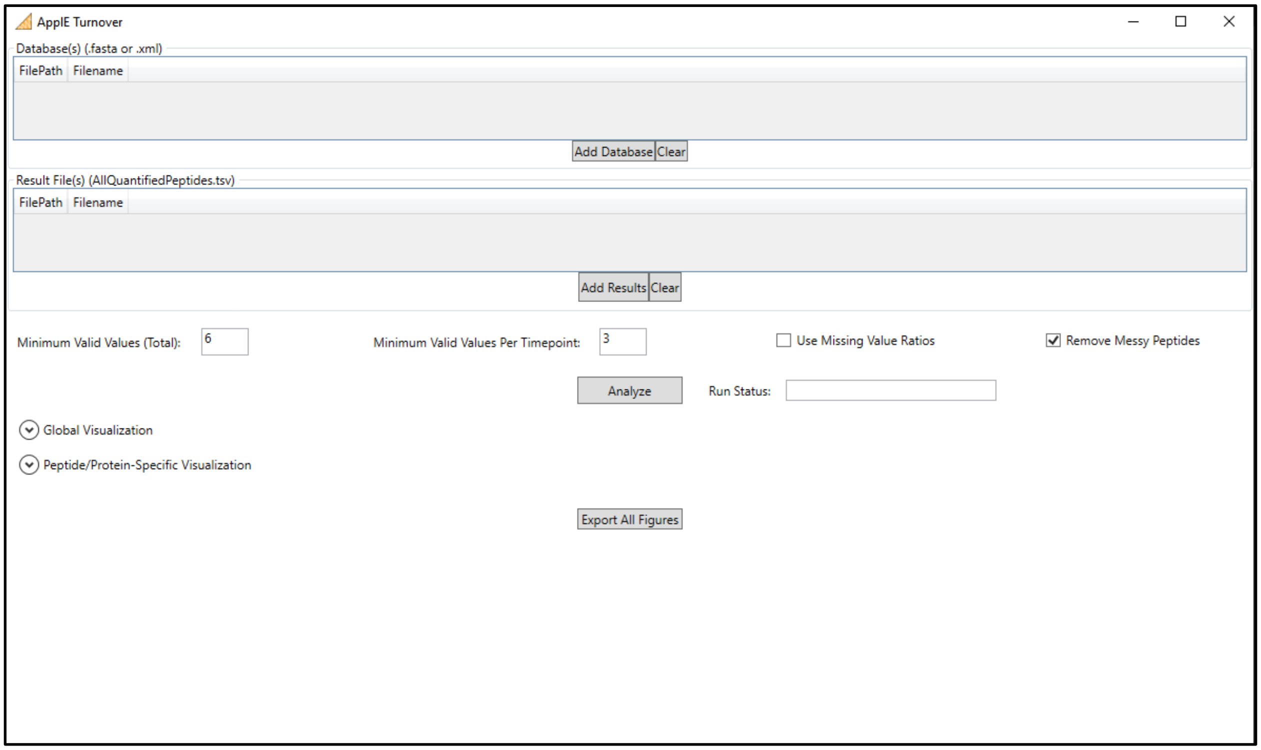

2.7. Drag and drop the "VisualizationFiles" folder from File Explorer onto the Apple Turnover screen. If successful, this will populate the "Result File(s)" table.

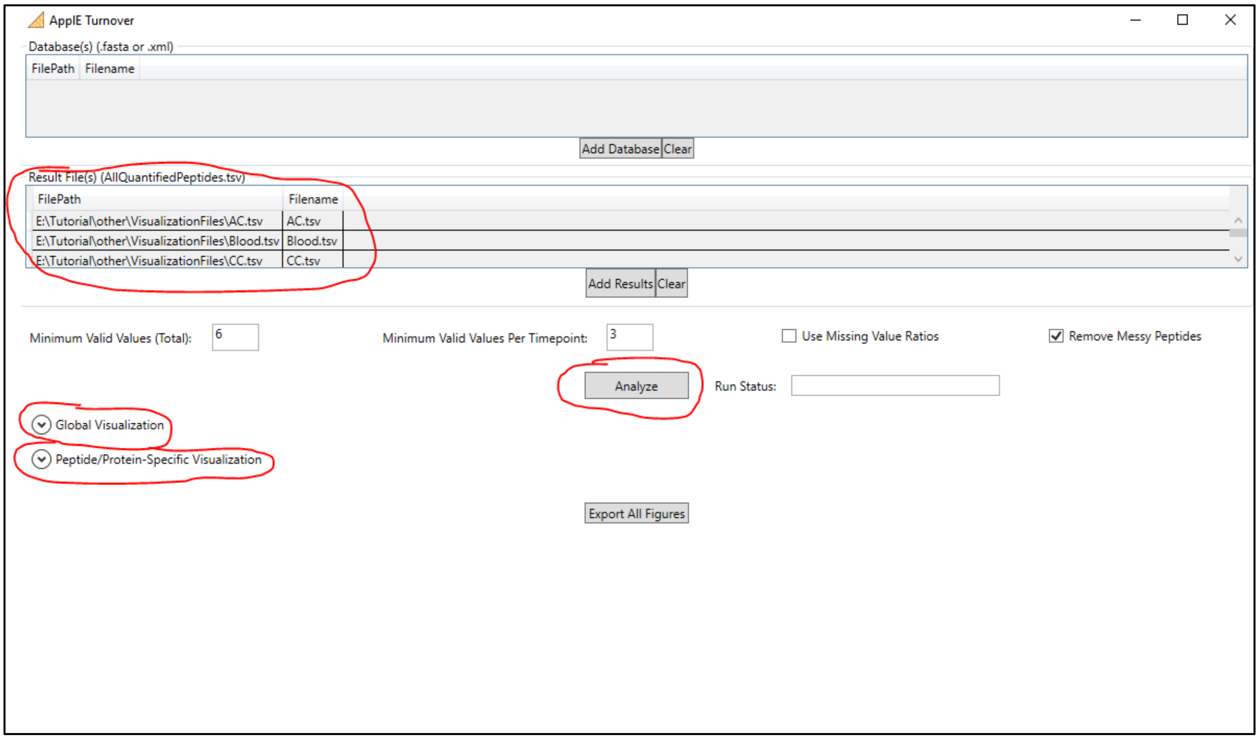

2.8. Click "Analyze". The process should take <10 seconds, and Run Status will say "Finished!"

2.9. Click on "Global Visualization" to see general trends for each tissue. Simply select the tissue of interest on the left panel to populate the two plots to the right. Note that the far-right plot sometimes "freezes". It can be refreshed by simply clicking on the plot.
The center plot shows the general fit of the model to the data for this particular tissue. The right plot displays a histogram of all measured half-lives. The data displayed is for peptides by default, and can be changed to proteins using the "Show Protein Data" button.
All plots have interactive capabilities. You can left-click and drag to move the plot or right-click and drag to zoom in and out.

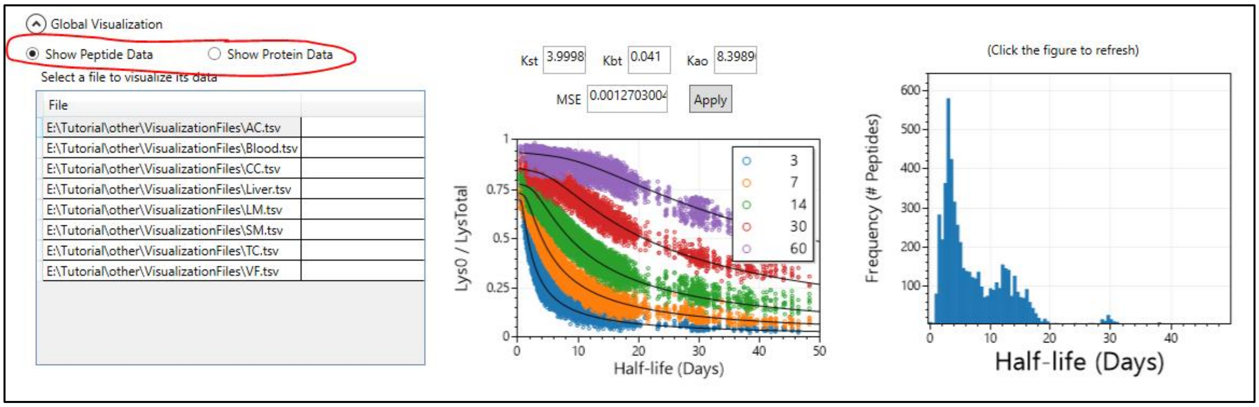

2.10. Click on “Peptide/Protein-Specific Visualization” to see data supporting specific peptide and protein half-lives. You can click on a peptide in the left table to display its data on the right. Search boxes in the top-left allow you to quickly look up a protein or peptide using its Uniprot accession number or peptide sequence.

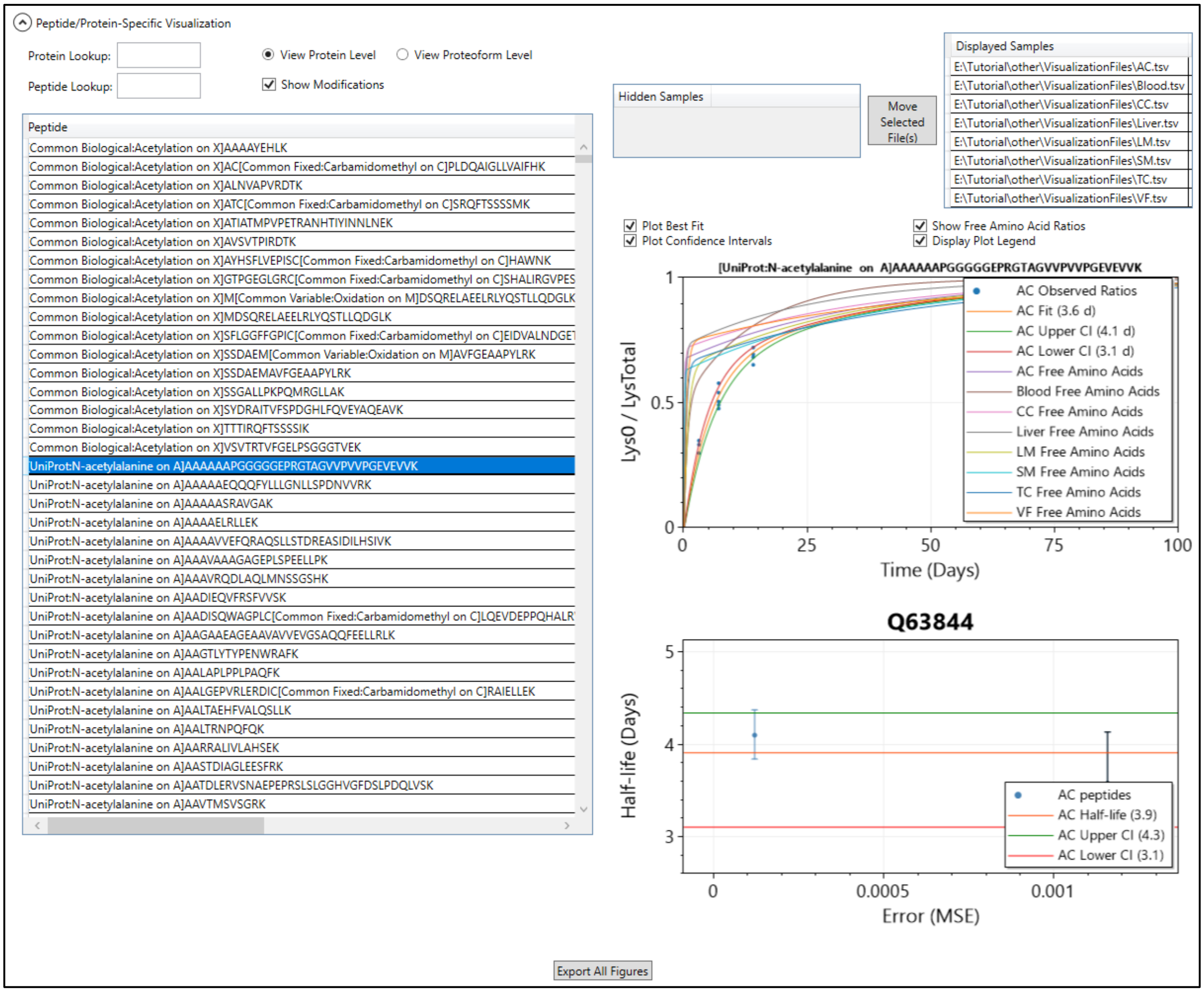

The top-right table allows you to select which tissues you want to visualize. All tissues are selected by default. To hide a tissue, simply click on the tissue in the top right and click “Move Selected File(s)”.

The top-right plot shows ratios and fits for the peptide selected on the left. The bottom-right plot shows all peptide information for the protein selected on the left.
Figures can be customized using the checkboxes in the top-right.

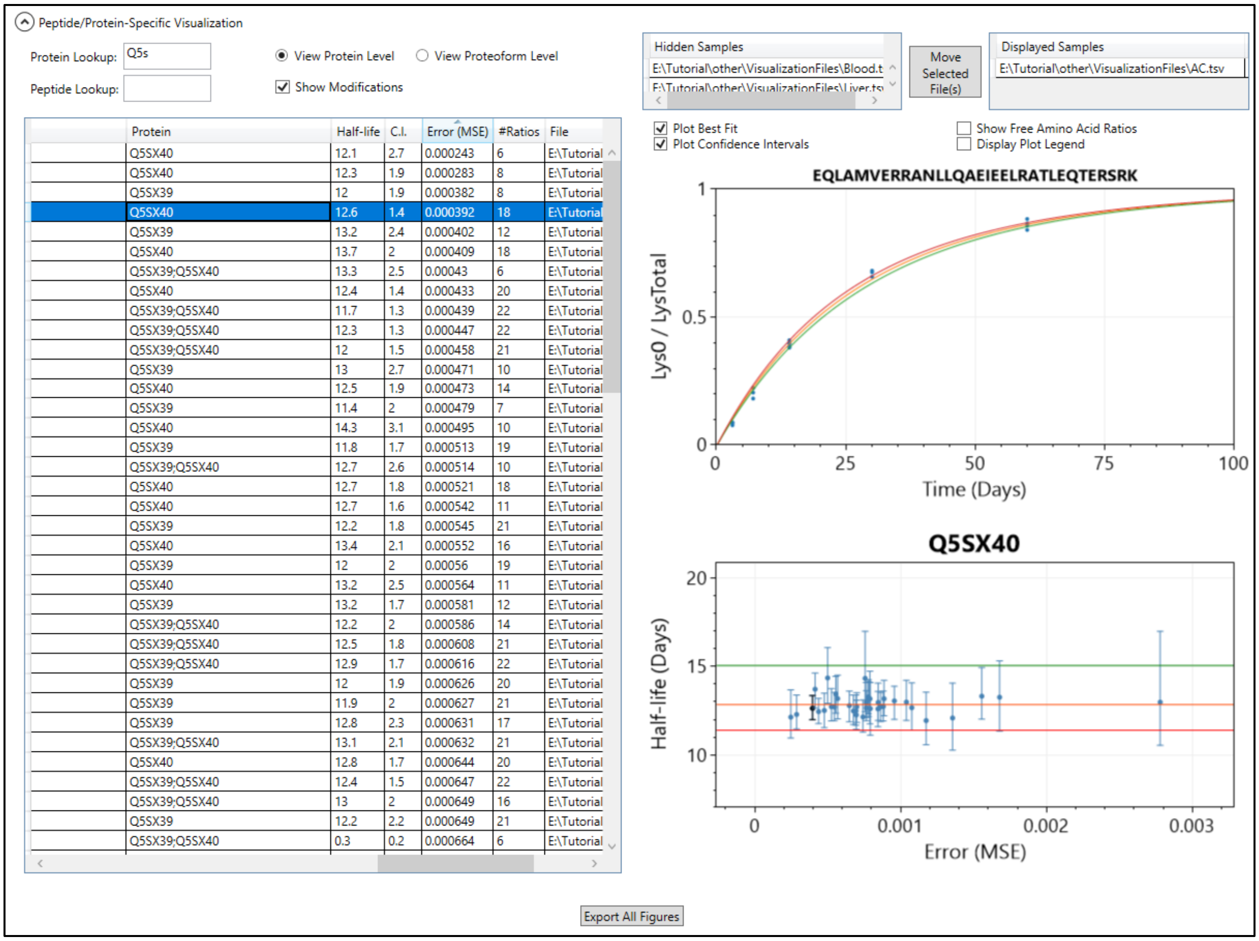

Figures can be exported by clicking on “Export All Figures” at the bottom of the page. Figures are automatically saved in the same folder as the displayed data.

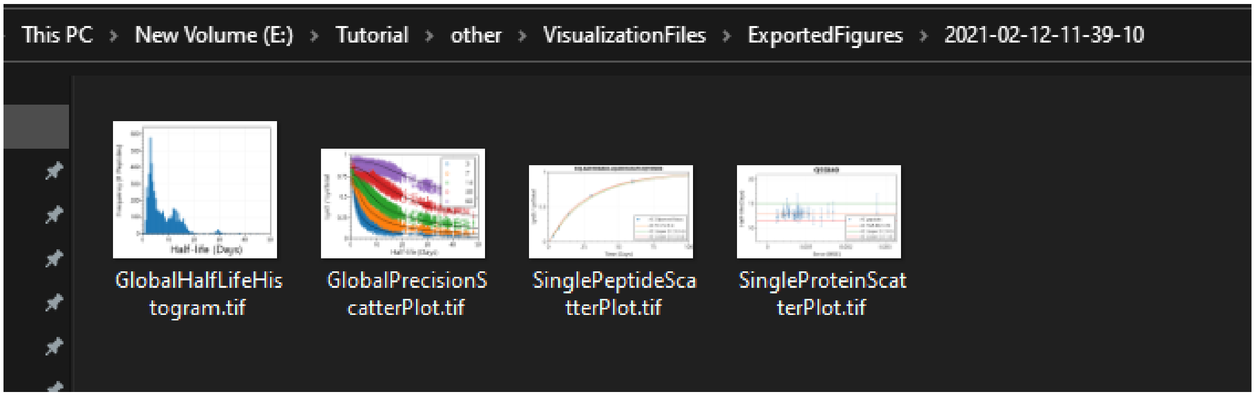

3. ANALYZE DATA WITH APPLE TURNOVER

The previous steps explained how to access and visualize the output of ApplE Turnover. If you have a new tissue or would like to reanalyze our data, ApplE Turnover is able to calculate half-lives and confidence intervals when provided with the protein database used in the database search, as well as the list of confidently identified peptides and the intensities of their labeled and unlabeled forms. These files for this resource can be found in MassIVE below.

3.1. Return to the ftp client (e.g. FileZilla).

3.2. Navigate to updates -> 2021-02-11_zrolfs_27533473

3.3. Drag and drop the folders “quant” and “sequence”.

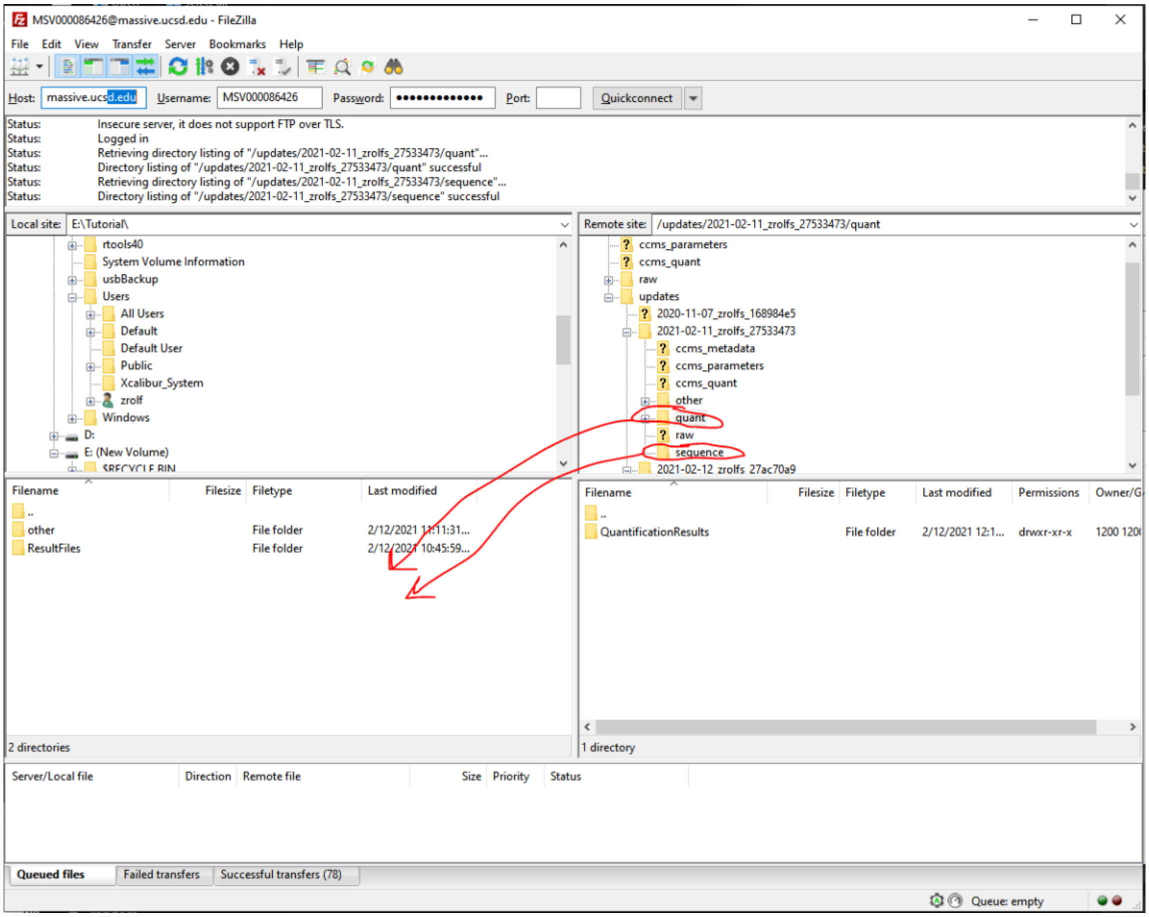

3.4. If there are Results Files in ApplE Turnover (e.g. from the Visualization tutorial above), then hit the “Clear” button below the file list.

3.5. Drag and drop the “quant” and “sequence” folders from File Explorer into ApplE Turnover and press “Analyze”. This will take about 20-40 minutes depending on your CPU.

3.6. After analysis, the result files will be output by ApplE Turnover (see step 1.8). These data can then be visualized in Apple Turnover (see step 2.9).

If you have any questions/comments, please reach out by opening an issue on our GitHub: https://github.com/smith-chem-wisc/AppleTurnover/issues

Thank you for your interest in this resource!
