# Supplementary material for: An atlas of protein turnover rates in mouse tissues
Source: Nat Commun. 2021 Nov 26;12:6778. doi: 10.1038/s41467-021-26842-3 (PMC8626426; doi:10.1038/s41467-021-26842-3)
Supplement: Supplementary file 2 — Description of Additional Supplementary Files [file 41467_2021_26842_MOESM2_ESM.docx]

**Description of Additional Supplementary Files**

**File Name:** Supplementary Data 1

**Description:** GO analysis of blood proteins.

**File Name:** Supplementary Data 2

**Description:** GO analysis of longer-lived cartilage proteins.

**File Name:** Supplementary Data 3

**Description:** Summary of differentially expressed proteoforms arising from post-translational

modifications.
