# Supplementary material for: An atlas of protein turnover rates in mouse tissues
Source: Nat Commun. 2021 Nov 26;12:6778. doi: 10.1038/s41467-021-26842-3 (PMC8626426; doi:10.1038/s41467-021-26842-3)
Supplement: Supplementary file 6 — Reporting Summary [file 41467_2021_26842_MOESM6_ESM.pdf]

nature research

Corresponding author(s): Nathan V. Welham

Last updated by author(s): Oct 12, 2021

Reporting Summary

Nature Research wishes to improve the reproducibility of the work that we publish. This form provides structure for consistency and transparency in reporting. For further information on Nature Research policies, see our Editorial Policies and the Editorial Policy Checklist.

Statistics

For all statistical analyses, confirm that the following items are present in the figure legend, table legend, main text, or Methods section.

n/a	Confirmed	
☐	☒	The exact sample size (n) for each experimental group/condition, given as a discrete number and unit of measurement
☐	☒	A statement on whether measurements were taken from distinct samples or whether the same sample was measured repeatedly
☐	☒	The statistical test(s) used AND whether they are one- or two-sided<br>*Only common tests should be described solely by name; describe more complex techniques in the Methods section.*
☒	☐	A description of all covariates tested
☐	☒	A description of any assumptions or corrections, such as tests of normality and adjustment for multiple comparisons
☐	☒	A full description of the statistical parameters including central tendency (e.g. means) or other basic estimates (e.g. regression coefficient) AND variation (e.g. standard deviation) or associated estimates of uncertainty (e.g. confidence intervals)
☐	☒	For null hypothesis testing, the test statistic (e.g. F, t, r) with confidence intervals, effect sizes, degrees of freedom and P value noted<br>*Give P values as exact values whenever suitable.*
☒	☐	For Bayesian analysis, information on the choice of priors and Markov chain Monte Carlo settings
☒	☐	For hierarchical and complex designs, identification of the appropriate level for tests and full reporting of outcomes
☐	☒	Estimates of effect sizes (e.g. Cohen's d, Pearson's r), indicating how they were calculated

Our web collection on statistics for biologists contains articles on many of the points above.

Software and code

Policy information about availability of computer code

Data collection	All mass spectrometry data were collected using the Xcalibur software (v. 4.0) provided by ThermoScientific with their Orbitrap QE-HF mass spectrometer.
Data analysis	A customized fork of MetaMorpheus (v. 0.0.305) and mzLib (v. 1.0.470) was used to identify and quantify peptides and proteins from the mass spectrometry data, and the source code, executables, and settings are freely available: https://doi.org/10.5281/zenodo.5563101. ApplE Turnover (v. 1.01) was used to calculate peptide and protein half-lives, confidence intervals, and statistical testing; it is available at https://doi.org/10.5281/zenodo.5555092. The source code and executables for all software used in this resource are publicly available. See "Code Availability" in the manuscript for details.

For manuscripts utilizing custom algorithms or software that are central to the research but not yet described in published literature, software must be made available to editors and reviewers. We strongly encourage code deposition in a community repository (e.g. GitHub). See the Nature Research guidelines for submitting code & software for further information.

Data

Policy information about availability of data

All manuscripts must include a data availability statement. This statement should provide the following information, where applicable:

- Accession codes, unique identifiers, or web links for publicly available datasets
- A list of figures that have associated raw data
- A description of any restrictions on data availability

All acquired and analyzed data has been made available through the MassIVE repository using the dataset identifier MSV000086426 [https://massive.ucsd.edu/ProteoSAFe/dataset.jsp?accession=MSV000086426]. All files were searched against a UniProtKB/Swiss-Prot .xml protein database of Mus musculus accessed May 19, 2020, and this database is provided in MassIVE with the file name: f.MSV000086426/updates/2021-02-11_zrolfs_27533473/

sequence/200519_Mouse_Reviewed_XML.xml. A tutorial for users has been provided (Supplementary Note–Tutorial).

Field-specific reporting

Please select the one below that is the best fit for your research. If you are not sure, read the appropriate sections before making your selection.

☒ Life sciences ☐ Behavioural & social sciences ☐ Ecological, evolutionary & environmental sciences

For a reference copy of the document with all sections, see nature.com/documents/nr-reporting-summary-flat.pdf

Life sciences study design

All studies must disclose on these points even when the disclosure is negative.

Sample size	Five timepoints were chosen for this study, with five mice per timepoint, for a total sample size of 25 mice. This sample size allows for some missing quantification values (e.g., some peptides not observed in all 25 samples). The five replicate mice for each timepoint enables determination of the biological variance in our measurements. The five timepoints were chosen to capture a wide range of half-lives without sacrificing precision. The number of biological replicates chosen in this work gives this resource very good statistical power compared to most studies using expensive isotopic labeling, which often employ only three mice per timepoint and only three or four timepoints.
Data exclusions	One mouse died prior to reaching its designated timepoint and was excluded. Two mice were harvested earlier than their expected timepoints to check the progress of the protein turnover and then were excluded from the data analysis because variance could not be determined for those timepoints.
Replication	With the exception of the data exclusions noted above, five biological replicates were analyzed for each timepoint to ensure reproducibility of the experimental findings.
Randomization	Randomization is not relevant to this study. Each mouse was raised and harvested in a similar environment. There was no experimental intervention applied which would have impacted the isotopic ratios of a group or have required randomization.
Blinding	Blinding is not relevant to this study. The mass spectrometer used to acquire data is impartial, and the human researchers are unable to manually modify the isotopic ratios within the tissues.

Reporting for specific materials, systems and methods

We require information from authors about some types of materials, experimental systems and methods used in many studies. Here, indicate whether each material, system or method listed is relevant to your study. If you are not sure if a list item applies to your research, read the appropriate section before selecting a response.

Materials & experimental systems

n/a	Involved in the study
☒	☐ Antibodies
☒	☐ Eukaryotic cell lines
☒	☐ Palaeontology and archaeology
☐	☒ Animals and other organisms
☒	☐ Human research participants
☒	☐ Clinical data
☒	☐ Dual use research of concern

Methods

n/a	Involved in the study
☒	☐ ChIP-seq
☒	☐ Flow cytometry
☒	☐ MRI-based neuroimaging

Animals and other organisms

Policy information about studies involving animals; ARRIVE guidelines recommended for reporting animal research

Laboratory animals	NBSGW mice (Jackson Laboratory stock 026622) were obtained from the Humanized Mouse Core of the University of Wisconsin-Madison. The mice were maintained on the Lys8 food for three generations, and the third generation mice used in the study were 8-10 weeks old at time zero (8 male, 14 female). Five age-matched (8-10 week old) and unlabeled C57/BL6J mice (2 male, 3 female; Jackson Laboratory stock 000664) were used as controls.
Wild animals	The study did not involve wild animals.
Field-collected samples	The study did not involve samples collected from the field.
Ethics oversight	The animal experiments were performed with approval of the Animal Care and Use Committee of the University of Wisconsin School of Medicine and Public Health.

Note that full information on the approval of the study protocol must also be provided in the manuscript.
